# The Histone H3K27 Demethylase UTX Regulates Synaptic Plasticity and Cognitive Behaviors in Mice

**DOI:** 10.3389/fnmol.2017.00267

**Published:** 2017-08-24

**Authors:** Gang-Bin Tang, Yu-Qiang Zeng, Pei-Pei Liu, Ting-Wei Mi, Shuang-Feng Zhang, Shang-Kun Dai, Qing-Yuan Tang, Lin Yang, Ya-Jie Xu, Hai-Liang Yan, Hong-Zhen Du, Zhao-Qian Teng, Feng-Quan Zhou, Chang-Mei Liu

**Affiliations:** ^1^State Key Laboratory of Stem Cell and Reproductive Biology, Institute of Zoology, Chinese Academy of Sciences Beijing, China; ^2^Savaid Medical School, University of Chinese Academy of Sciences Beijing, China; ^3^School of Life Sciences, University of Science and Technology of China Hefei, China; ^4^The State Key Laboratory of Integrated Management of Pest Insects and Rodents, Institute of Zoology, Chinese Academy of Sciences Beijing, China; ^5^Department of Orthopaedic Surgery, The Johns Hopkins University School of Medicine Baltimore, MD, United States; ^6^The Solomon H. Snyder Department of Neuroscience, The Johns Hopkins University School of Medicine Baltimore, MD, United States

**Keywords:** *Utx*, H3K27me3, synaptic transmission, cognition

## Abstract

Histone demethylase UTX mediates removal of repressive trimethylation of histone H3 lysine 27 (H3K27me3) to establish a mechanistic switch to activate large sets of genes. Mutation of *Utx* has recently been shown to be associated with Kabuki syndrome, a rare congenital anomaly syndrome with dementia. However, its biological function in the brain is largely unknown. Here, we observe that deletion of *Utx* results in increased anxiety-like behaviors and impaired spatial learning and memory in mice. Loss of *Utx* in the hippocampus leads to reduced long-term potentiation and amplitude of miniature excitatory postsynaptic current, aberrant dendrite development and defective synapse formation. Transcriptional profiling reveals that *Utx* regulates a subset of genes that are involved in the regulation of dendritic morphology, synaptic transmission, and cognition. Specifically, *Utx* deletion disrupts expression of neurotransmitter 5-hydroxytryptamine receptor 5B (*Htr5b*). Restoration of *Htr5b* expression in newborn hippocampal neurons rescues the defects of neuronal morphology by *Utx* ablation. Therefore, we provide evidence that *Utx* plays a critical role in modulating synaptic transmission and cognitive behaviors. *Utx* cKO mouse models like ours provide a valuable means to study the underlying mechanisms of the etiology of Kabuki syndrome.

## Introduction

Accumulating evidence suggests that epigenetic regulations play critical roles in neurological disorders (Tsankova et al., 2007; Ma et al., 2010). Trimethylation at Lysine 27 of histone H3 (H3K27me3) establishes a repressive chromatin state in silencing an array of crucial genes, which contributes to important biological processes including X-inactivation, genomic imprinting, stem cell maintenance, circadian rhythms, and cancer (Plath et al., 2003; Etchegaray et al., 2006; Sparmann and van Lohuizen, 2006; Van der Meulen et al., 2014). In mammals, the dynamic steady-state levels of dimethylation and trimethylation of histone H3 lysine 27 (H3K27me2/3) are mainly maintained through balance between methyltransferase Polycomb Repressor Complex 2 (PRC2) and the demethylases UTX (also known as KDM6A) and Jumonji D3 (JMJD3, also known as KDM6B) (Agger et al., 2007; Lee et al., 2007; Van der Meulen et al., 2014). H3K27me3 is involved not only in the balance between self-renewal and differentiation of neural stem cells (NSCs) (Pereira et al., 2010; Zhang et al., 2014), but also in the development of neurodegenerative diseases (Li et al., 2013; von Schimmelmann et al., 2016).

The *Utx* gene is encoded on the X chromosome but escapes X inactivation in females and is ubiquitously expressed (Greenfield et al., 1998). Earlier studies have demonstrated a critical role of *Utx* in cell reprogramming, cell differentiation, embryonic development, muscle regeneration, circadian rhythm, and senescence (Agger et al., 2007; Mansour et al., 2012). *Utx* mutation has been found in a variety of human cancers, including multiple myeloma, esophageal, renal cancer, bladder cancer, and T-cell acute lymphoblastic leukemia (van Haaften et al., 2009; Van der Meulen et al., 2014). Interestingly, recent studies have shown that *de novo* deletion and point mutations of *Utx* are associated with Kabuki syndrome (Lederer et al., 2012; Miyake et al., 2013), a rare congenital anomaly syndrome with mild to severe intellectual disability, growth retardation, and a variety of visceral malformations. Recent clinical data suggests that UTX and UTX-mediated H3K27me2/3 demethylation may play a critical role in the brain development (Miyake et al., 2013). Although *Utx* is highly expressed in most of the brain regions (Xu et al., 2008), its functional role in the central nervous system (CNS) is largely unknown. More importantly, we still do not know whether deletion of UTX in brain could replicate moderate-to-severe congenital anomaly/mental retardation observed from clinical Kabuki syndrome patients, and the pathomechanisms of Kabuki syndrome as well as the roles of *Utx* in the brain are largely unknown.

To investigate the function of *Utx* in CNS, we generated forebrain specific *Utx* deletion mice (cKO). Here we show that *Utx* cKO mice exhibited anxiety-like behaviors, learning and memory impairments. At the physiological and cellular levels, these cKO mice displayed abnormal synaptic transmission and long-term potentiation (LTP) accompanied with the abnormal neuronal morphology. Bioinformatics analysis revealed that UTX mediated-H3K27me3 demethylation suppressed expression of a subset of genes that are involved in the regulation of neuronal morphology and synaptic activity. The neurotransmitter 5-hydroxytryptamine receptor 5B (*Htr5b*) is a downstream target of *Utx*. Overexpression of *Htr5b* can rescue neuronal morphological impairment induced by *Utx* deficiency. The results from the present study provide evidence for the first time that *Utx* plays important roles in neuroplasticity and behaviors. Our work also suggests that *Utx* deficiency lead to cognition deficits underlying intellectual disability in Kabuki syndrome.

## Materials and methods

### Mice

All experiments involving animals were performed in accordance with the animal protocol approved by the Institutional Animal Care and Use Committee at the Institute of Zoology, Chinese Academy of Sciences. Mice were housed in groups of 3–5 animals in a 12 h light/12 dark cycle, with standard mouse chow and water *ad libitum*. *Utx*^f/f^ (stock number 021926), transgenic *Nestin-Cre* (stock number 003771), and *Emx1-Cre* transgenic mice (stock number 005628) were bought from Jackson Lab. The conditional *Utx* knockout mice were generated by breeding *Utx*^f/f^ mice with either *Nestin-Cre* or *Emx1-Cre* transgenic mice.

### Behavioral tests

All behavioral tests were performed during the light cycle between 07:00 and 19:00. Male mice at 2–3 months of age were used for all the behavioral tests. All the videos were analyzed by the Smart software (Pan Lab, Harvard Apparatus).

### Open field test

The open field test was conducted in a large box (72 × 72 × 36 cm) in a brightly lit room. Mice were placed in the center of the maze and were monitored from above by a video camera. The number of entries in the center zone (18 × 18 cm) of the maze was recorded over a 5-min trial to evaluate anxiety effects.

### Light-dark box test

Light-dark box test was performed as previously described (Costall et al., 1993). An apparatus (45 × 27 × 27 cm) consisting of two chambers, a black chamber (18 × 27 cm) and a light chamber (27 × 27 cm), was used for the light/dark exploration test. Mice were placed into the dark box and allowed to move between the light box and dark box for 5 min. The total number of transitions and the time spent in each box were analyzed.

### Elevated plus maze

The elevated plus maze is a plus-shaped apparatus with four arms (two open, 62 × 8.5 cm; two closed, 62 × 8.5 × 30 cm), elevated 70 cm from the floor. Mice were placed at the junction of the four arms of the maze, and allowed to freely explore the maze for 5 min. The number of entries and duration in each arm were analyzed (Rodgers and Dalvi, 1997).

### Morris water maze

Morris water maze test was performed as described previously (Vorhees and Williams, 2006). A 120 cm diameter, 45 cm deep Morris water maze was filled with water to a depth of 25 cm. Target escape platform (diameter 13 cm) was hidden 1.5 cm beneath the surface of the water at the center of a given quadrant of the water tank. Four extra-maze cues, in different shapes, colors, and sizes, were uniformly located on the wall surrounding the water tank. The water temperature was adjusted to 21 ± 1°C. During training trials, mice were trained in four trials per day starting from different sites. The mice were allowed to swim for up to 1 min to locate the platform. If it failed to locate the platform within that time, escape was assisted. Mice were introduced gently to the hidden platform and allowed to rest on the platform for 15 s. For the probe trial, 24 h after the final training day the platform was removed and time spent and entry in each of the four quadrants were recorded.

### Electrophysiology

#### Acute hippocampal slice preparation

Acute hippocampal slices were prepared from 8-week-old cKO mice and their WT littermates. Briefly, mice were deeply anesthetized with isoflurane and decapitated. The brain was rapidly removed and transferred into ice-cooled artificial cerebrospinal fluid (aCSF) (in mM: 125 NaCl, 2.5 KCl, 1.25 NaH_2_PO_4_, 25 NaHCO_3_, 25 D-Glucose, 2 CaCl_2_, and 1.5 MgCl_2_ saturated with 95% O_2_ and 5% CO_2_ to pH: 7.4). A filter paper was placed on the bottom of a petri dish in advance. The brain was transferred into the petri dish containing ice-cold aCSF. Coronal slices with 300 μm thickness containing hippocampus were cut with a vibratome (Leica, VT 1000 S, Germany), which was filled with the cold aCSF. The prepared slices were incubated in oxygenated aCSF at room temperature at least for 1 h, and then individual slices were transferred to a recording chamber, which was bubbled with oxygenated aCSF. The temperature of the aCSF in the recording chamber was 31 ± 1°C. Individual pyramidal neurons visualized with an Olympus microscope (Olympus BX50-WI, Olympus, Japan) fitted with a 40x long-working distance objective (NA 0.8).

#### Electrophysiological recordings

Whole-cell patch-clamp recordings were made using an Axopatch 700B amplifier and the pClamp10.6 software was used for data acquisition and analysis. Patch pipettes (4–6 MΩ) were pulled from borosilicate glass capillaries (GB 150F-8P) with a micropipette puller (Sutter instrument, USA). The internal pipette solution contained: (in mM) 135 K-gluconate, 10 HEPES, 2 MgCl_2_, 10 EGTA, 0.3 MgGTP, 0.5 Na_2_ATP (pH 7.3 with KOH). Spontaneous miniature excitatory postsynaptic currents (mEPSCs) were recorded under the whole-cell configuration of voltage clamp. The membrane potential was held at −70 mV. Series resistances and cell capacitance compensation were carried out prior to recording. The recordings were included only in those with high resistance seal (>1 GΩ) and a series resistance <25 MΩ. To isolate AMPA receptor-mediated mEPSCs, 10 nM glycine, 10 μM bicuculline (the GABAA receptor antagonist), and D-AP-5(NMDA receptor antagonist) were added to the aCSF. In addition, TTX (0.5 μM) was included in the extracellular solution.

Field excitatory postsynaptic potentials (fEPSPs) were recorded in the CA1 region of hippocampus. A bipolar concentric stimulating electrode (FHC Inc., Bowdoin, ME) was placed at the Schaffer collaterals to deliver stimuli. A glass recording electrode (3–4 MΩ) filled with aCSF was positioned in the stratum radiatum of the CA1 area. fEPSPs in CA1 were induced by stimulus at 0.033 Hz with an intensity that elicited a fEPSP amplitude of 40–50% of the maximum. After establishment of stable baseline recordings for at least 15 min, LTP was induced by a high-frequency stimulation (HFS) consisting of one train of 100 Hz stimulation for 1 s at baseline stimulation intensity. The fEPSP signals were digitized using Digidata1440A interface board. The data were sampled at 10 kHz and filtered at 2 kHz. Recordings were analyzed using the Clampfit 10.6 (Axon Instruments, Foster City, CA).

### Neuronal culture and transfection

Primary hippocampal neurons (1 × 10^4^ cells per well in a 24-well plate) were cultured from P0 *Utx* cKO and WT mice on plates coated with poly-D-lysine (100 μg/ml). The dissected hippocampus tissue was digested with trypsin-EDTA for 10 min at 37°C. The tissue was then washed three times with MEM plus 10% FBS and dissociated with the culture medium. Then neurons were grown in Neurobasal medium (Invitrogen) supplemented with 2% B27 (Invitrogen) and 2 mM GlutaMAX (Invitrogen) and penicillin/streptomycin. Primary hippocampal neurons were transfected with Lipofectamine 2000 (Invitrogen) according to the manufacturer's protocol.

### Neuronal morphology analysis

#### *In vivo* dendritic and spine density analysis

We analyzed *in vivo* dendrites and spine density by using Golgi Stain Kit following the manufacturer's instructions. Generally, freshly 8-week-old mouse brains were incubated in the dark in Golgi solution A+B (FD Rapid Golgi Stain Kit, PK401, FD NeuroTechnologies) for 2 weeks. After incubation, brains were transferred into Solution C and were stored at room temperature in the dark for 3 days. Coronal sections (200 μm) were cut with a Leica CM1950 cryostat and mounted on 3% gelatin-coated slides. Staining procedures were followed according to the manufacturer's protocol (FD NeuroTechnologies), and slides were dehydrated in ethanol and mounted with Permount for microscopy. Images of the dendrites and the second segment apical dendrite spine were acquired on LSM 710 confocal microscope with 20 × and 100 × oil lense, respectively. Dendritic branches were traced, and their lengths were calculated using the Simple Neurite Tracer plugin of Fiji.

#### *In vitro* dendritic and spine analysis

For dendrites analysis, cultured neurons were fixed with 4% paraformaldehyde and then were washed with PBS at 7 day *in vitro* (DIV-7). Fixed neurons were blocked by 2% normal goat serum and 0.1% Triton X-100 in TBS for 1 h at room temperature. Neurons were incubated in primary antibodies (Map2, Mab3418, Millipore, 1:1,000) overnight at 4°C, and then incubated with secondary antibodies. Images of dendrites were acquired on Zeiss LSM710 confocal microscope with a 20 × lense. For spine analysis, cultures were used for immunostaining at DIV-19. Images of spines were acquired on Zeiss LSM710 confocal microscope with a 63 × oil lense. The secondary dendritic spines were analyzed.

### Stereotactic injection

Stereotactic injections into the hippocampus (stereotaxic coordinates from Bregma: 2.0 mm caudal, 1.2 mm lateral, 2.0 mm ventral; 2.8 mm caudal, 2.0 mm lateral, 1.7 mm ventral) were performed as follows: 8-week-old *Utx*^flox/flox^ were bilaterally injected with 1 μl Adeno-associated virus (AAV) 2/8 (Heyuan, China; Titer: Control virus:6.24 × 10^12^ V.G./ml; Cre virus: 5.04 × 10^12^ V.G./ml) at a rate of 0.125 μl/min.

### RNA isolation and RT-qPCR

Total RNA were extracted from hippocampus tissues or cultured cells according to procedures using Trizol reagent (Invitrogen). Two micrograms of total RNA was reverse transcribed using either oligo (dT) primers or specific primers by using a Transcriptor First Strand cDNA Synthesis Kit (Roche). For real-time PCR analysis, according to the manufacturer's instructions, using a SYBR mix from Roche. 25 ng of cDNA and 0.5 mM primers were used in a final volume of 20 μl. The PCR steps were performed 30 s pre-denaturation at 95°C, followed by 45 cycles of 10 s denaturation at 94°C, 30 s annealing at 60°C, 30 s extension at 72°C. The analysis of RT-qPCR used the 2^−^^ΔΔC_T_^ method. Each reaction was run in triplicate and analyzed following the ^ΔΔ^Ct method using glyceraldehyde-3-phosphate dehydrogenase (GAPDH) as a normalization control. All primers are listed in Table 1.

**Table 1:** Description of primers used in this study.

**Gene/Primer**	**Primer sequence (5′–3′)**
Utx-F	CGGGCGGACAAAAGAAGAAC
Utx-R	CATAGACTTGCATCAGATCCTCC
Uty-F	GGAATGAATGTGTTCCATGTCT
Uty-R	CTCATGTAGACCAAGATGACC
Htr5b-F	CTGGTGAGCGAGTTGTCCG
Htr5b-R	GCGTGATAGTCCAGTAGCGA
Egr1-F	AGCGAACAACCCTATGAGCACC
Egr1-R	ATGGGAGGCAACCGAGTCGTTT
Egr3-F	CTGACAATCTGTACCCCGAGGA
Egr3-R	GCTTCTCGTTGGTCAGACCGAT
Shank1-F	CAACCATCTCCCTGCGTTCCAA
Shank1-R	GAGAGCCATCTGATACACGGTC
Wnt4-F	GAGAACTGGAGAAGTGTGGCTG
Wnt4-R	CTGTGAGAAGGCTACGCCATAG
Nos1-F	CTACACCACACACATCCTCAAGG
Nos1-R	GCACTTTGGAGAGCGGGCAATA
Edn1-F	CTACTTCTGCCACCTGGACATC
Edn1-R	CGCACTGACATCTAACTGCCTG
Actn2-F	CACCTGGAGTTTGCCAAGAGAG
Actn2-R	GCCTTGAACTGCTCATGTGCAG
Cacna1g-F	GACCATGTGGTCCTCGTCATCA
Cacna1g-R	TTTCAGCCAGGAAGACTGCCGT
Tgfb2-F	TTGTTGCCCTCCTACAGACTGG
Tgfb2-R	GTAAAGAGGGCGAAGGCAGCAA
Gpr88-F	TCCTCCACTTCGACCTCCAC
Gpr88-R	GCCCGAGTACAGGAGAGAC

### Protein quantification

Hippocampal tissues and cultured neurons were lysed in buffer containing 25 mM HEPES at pH7.9, 150 mM NaCl, 1 mM PMSF, 20 mM NaF, 1 mM DTT, 0.1% NP40, and proteinase inhibitor cocktails (Roche). Protein concentrations were determined by Folin phenol method with bovine serum albumin as standard. Twenty micrograms of the protein was separated on 8–12% SDS-PAGE gels (Bio-Rad) and transferred to PVDF membranes (Millipore). The membranes were blocked in 5% BSA in TBS-T with 0.05% Tween-20 and incubated with primary antibodies at 4°C overnight. Dilutions of primary antibodies were 1:1000 for UTX (E409, Millipore), H3K27me3 (1:1,000, 07449, Millipore), PSD95 (ab2723, Abcam), Synapsin (ab8049, Abcam), and 1:10,000 for β-actin antibody (Sigma). As for the secondary antibodies, we used HRP-linked goat anti-mouse or HRP-Linked goat anti-rabbit at 1:500. Enhanced chemoluminescence (ECL, Pierce) was used for detection. Quantification of the blots was determined with Quantity One Ver.4.4.0 (BioRad, USA).

### Immunohistochemistry

Adult mice were anesthetized, perfused with 4% PFA. Brain tissue was dissected out, equilibrated in 30% sucrose, and sectioned into 40 μm-thick serial sections. The brain sections were washed in PBS for 15 min three times, and then blocked in blocking solution (3% BSA+0.3%Triton X-100+0.2% sodium azide) at room temperature for 1 h. The primary antibodies we used are as follows: anti-H3K27me3 (1:1,000, 07449, Millipore), anti-Map2 (1:1,000, Mab3418, Millipore), anti-GFP (1:1,000, A10262, Life technology), anti-NeuN (1:1,000, millipore), anti-Doublecortin (1:500, millipore). After incubation in primary antibody solution at 4°C overnight, the brain sections were washed with TBS for 30 min three times and then incubated with the secondary antibodies conjugated to Alexa Fluor 488 or 594 with a concentration of 1:500 at room temperature. The sections were finally stained with DAPI and mounted using adhesion anti-fade medium.

### Bioinformatics analyses

Only transcripts that showed more than 1.5-fold differential expression compared to control were subjected to relevance network analysis. GO analysis was performed by Database for Annotation, Visualization and Integrated Discovery (DAVID v6.7) (Huang et al., 2009). Mouse phenotype and gene enrichment were analyzed by WEB-based Toolkit (https://toppgene.cchmc.org/).

### Statistical analysis

Statistical analysis was performed using SPSS 17.0 software (SPSS Inc., Chicago, IL, USA) with one-way or two-way analysis of variance as specified in legend of each figure. Prior to all statistical analyses, data were examined for normality of variance using the Kolmogorov-Smirnov test. All data were presented as mean ± SEM, and statistically significant was defined as ^*^p < 0.05; ^**^p < 0.01; ^***^p < 0.001.

## Results

### *Utx* cKO mice display anxiety-like behaviors and spatial learning and memory deficits

**Figure 1:**
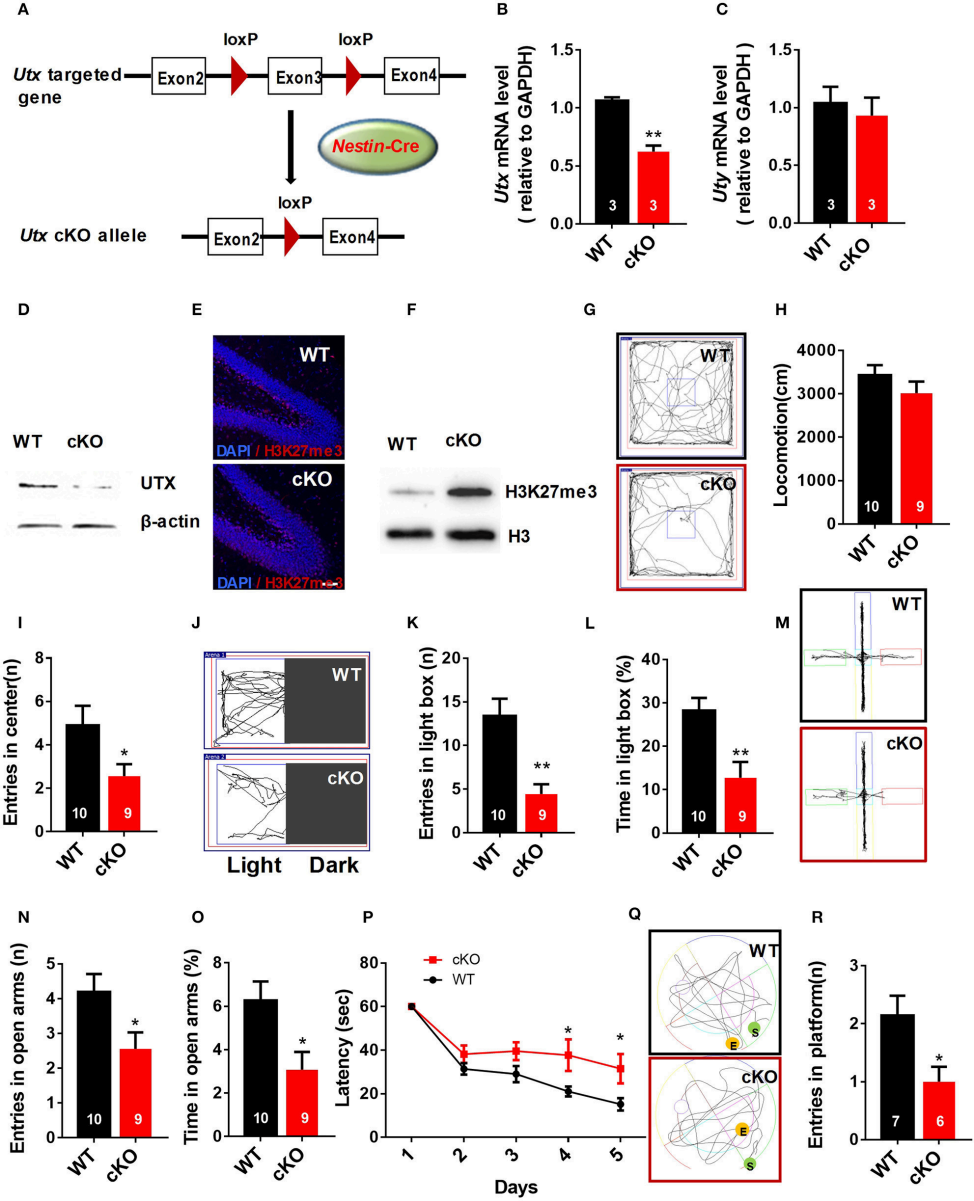
*Nestin*-Cre *Utx* cKO mice display anxiety-like behaviors and spatial learning and memory deficits. **(A)** Strategy for the generation of *Nestin*-Cre *Utx* cKO mice. **(B)** Real-time PCR analysis showing that *Utx* mRNA is decreased in the hippocampus of cKO mice (*n* = 3, *p* < 0.01). **(C)** Real-time PCR analysis showing that *Uty* mRNA had no change in the hippocampus of cKO mice (*n* = 3, *p* = 0.6287). **(D)** Western blotting analysis confirmed knockout of *Utx* in the hippocampus of cKO mice. **(E)** Representative images of fluorescent immunohistochemistry showing an increase of H3K27me3 in the dentate gyrus of hippocampus in cKO mice. Scale bars, 50 μm. **(F)** Protein levels of H3K27me3 are increased in the hippocampus from cKO mice. **(G)** Representative trajectory maps in an open field test. **(H)** *Utx* cKO mice had comparable locomotivity to WT littermate mice in open field test over a 5-min period [WT, *n* = 10; cKO, *n* = 9; two-tailed *t*-test: *t*_(17)_ = 1.181, *P* = 0.2539]. **(I)** cKO mice showed decreased entry into the center zone during 5-min open field test [WT, *n* = 10; cKO, *n* = 9; two-tailed *t*-test: *t*_(17)_ = 2.157; *P* = 0.0456]. **(J)** Representative trajectory maps of the light-dark box test. **(K)** cKO mice had decreased entry into the light box during 5-min light-dark box test [WT, *n* = 10; cKO, *n* = 9; two-tailed *t*-test: *t*_(17)_ = 3.855, *P* = 0.0013]. **(L)** cKO mice spent less time in the light box over a 5-min light-dark box test [WT, *n* = 10; cKO, *n* = 9; two-tailed *t*-test: *t*_(17)_ = 3.423, *P* = 0.0032]. **(M)** Representative trajectory maps of WT and *Utx* CKO mice in the elevated plus maze test. **(N)** cKO mice showed decreased entry into the open arms during 5-min elevated plus maze test [WT, *n* = 10; cKO, *n* = 9; two-tailed *t*-test: *t*_(17)_ = 2.338, *P* = 0.0318]. **(O)** cKO mice spent less time significantly in the open arms over a 5-min elevated plus maze compared to WT littermates [WT, *n* = 10; cKO, *n* = 9; two-tailed *t*-test: *t*_(17)_ = 2.659, *P* = 0.0165]. *P* cKO mice spent more time in reaching the platform during 5-day training in Morris water maze test [WT, *n* = 7; cKO, *n* = 6; repeated measures analysis of variance (ANOVA), followed by *Turkey post-hoc* test; Group effect: *F*_(1, 11)_ = 8.977, *P* = 0.012; Day4: *t*_(11)_ = 2.337, *P* = 0.039; Day5: *t*_(11)_ = 2.366, *P* = 0.037]. **(Q)** Representative trajectory maps of WT and *Utx* CKO mice in water maze test. **(R)** cKO mice showed less platform crossing in Morris water maze test [WT, *n* = 7; cKO, *n* = 6; two-tailed *t*-test: *t*_(11)_ = 2.602, *P* = 0.0246]. **(P)**, repeated measures analysis of variance (ANOVA), followed by *Turkey post-hoc* test; others, unpaired *t*-test. n, number of mice. ^*^P < 0.05; ^**^P < 0.01. Error bars, s.e.m.

To evaluate the function of *Utx* in the brain, we used *Utx* conditional knockout (cKO) mice that lack *Utx* expression in NSCs during development (Figure 1A). *Utx* cKO male mice could survive to adult, however, almost all *Utx* cKO female mice died within 3 weeks after birth. *Utx* deletion was then confirmed at both mRNA and protein levels in cKO mice (Figures 1B,D), and we did not observe any change of its Y chromosome homolog Uty at mRNA level in the cKO brain (Figure 1C). Since *Utx* is a histone demethylase and functions in removal of repressive trimethylation of H3K27 (Agger et al., 2007), we next examined the histone marks H3K27me3 in cKO mice. Consistent with our expectations, *Utx* deletion significantly resulted in increased expression of H3K27me3 in the hippocampus compared to that in the WT littermates (Figures 1E,F).

Male *Utx* cKO mice and their WT littermates were subjected to a battery of behavioral tests including tests for anxiety-like behaviors as well as learning and memory. We firstly conducted open field test and found that *Utx* cKO mice had smaller entries into the center of the arena (Figures 1G,I), whereas total distance traveled was comparable to that of WT littermates (Figure 1H). Increased anxiety-like behaviors of cKO mice were then confirmed in the light-dark box test and the elevated plus maze test. In the light-dark box test, *Utx* cKO mice showed a substantially decreased entries (Figures 1J,K) and time spent (Figure 1L) in the light box. Similarly, *Utx* cKO mice displayed decreased entries (Figures 1M,N) and spent less time in the open arms when compared with the WT littermates in the elevated plus maze test (Figure 1O).

Next, we determined whether *Utx* cKO mice have deficits in spatial learning and memory using the Morris water maze test. We found that cKO mice take a longer time to locate the hidden platform in the training trials (Figure 1P) and spend less time in the platform zone during the probe test (Figures 1Q,R), indicating that *Utx* cKO mice have deficits in hippocampus-dependent spatial learning and memory.

**Figure 2:**
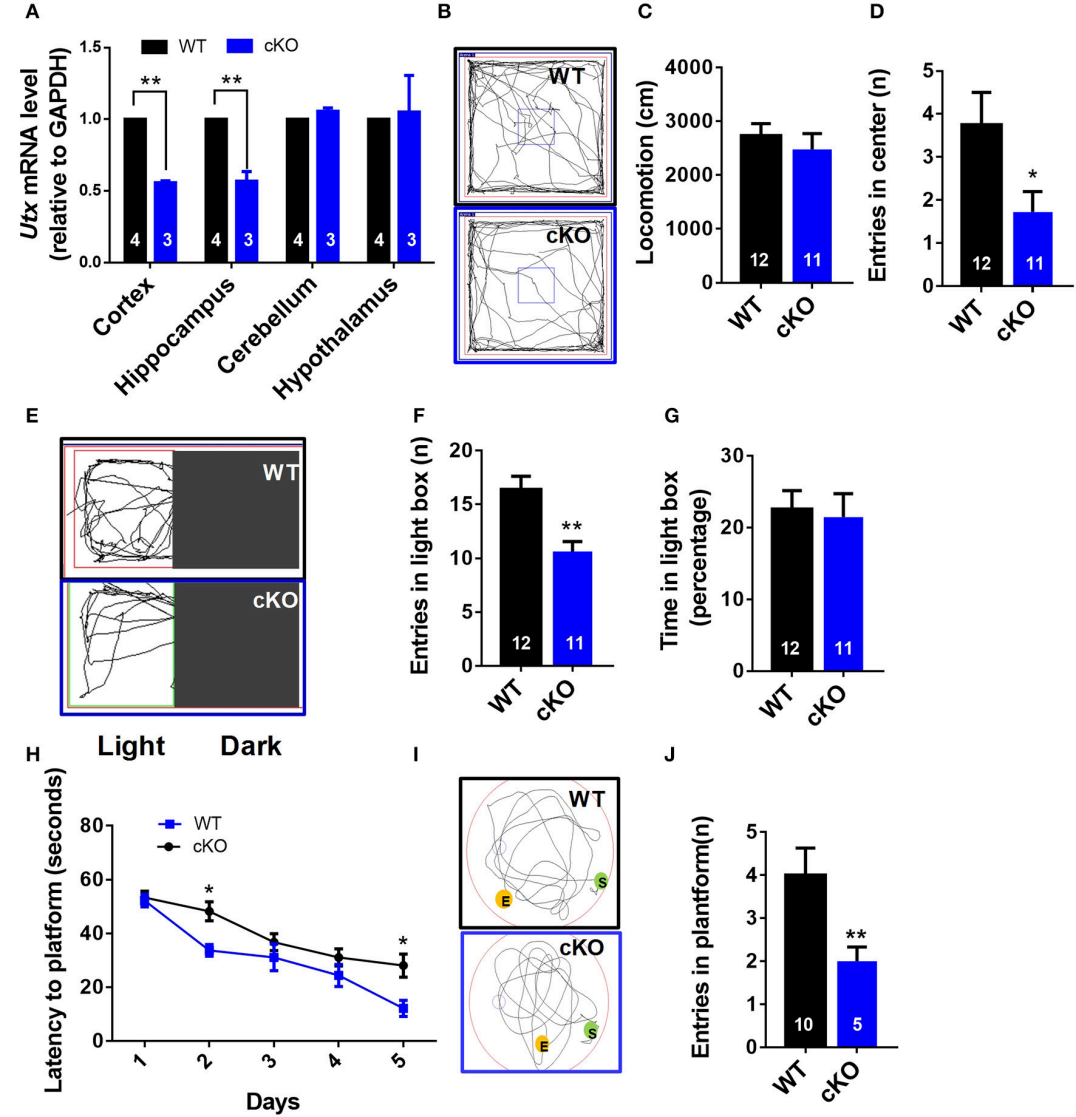
*Emx1*-cre *Utx* cKO mice display anxiety-like behaviors and congenital abnormities. **(A)** Real-time PCR analysis showing that *Utx* mRNA are decreased in the cortex and hippocampus in *Emx1-Cre Utx* cKO mice [WT, *n* = 4; cKO, *n* = 3; two-tailed *t*-test: *t*_(5)_ = 40.531, *P* < 0.001] and hippocampus [WT, *n* = 4; cKO, *n* = 3; two-tailed *t*-test: *t*_(5)_ = 7.610, *P* = 0.001]. **(B)** Representative trajectory maps of *Utx* cKO and WT mice in the open field test. **(C)** *Utx* cKO and WT mice displayed similar locomotivity in the open field test over a 5-min period [WT *n* = 12, cKO *n* = 11; two-tailed *t*-test; *t*_(21)_ = 0.7064; *P* = 0.4877 for WT vs. cKO]. **(D)** *Utx* cKO mice had decreased entries into the center zone during 5-min open field test [WT *n* = 12, cKO *n* = 11; two-tailed *t*-test; *t*_(21)_ = 2.238; *P* = 0.0362 for WT vs. cKO]. **(E)** Representative trajectory maps in the light-dark box test. **(F)** *Utx* cKO mice showed decreased entries into the light box during 5-min light-dark test [WT, *n* = 12; cKO, *n* = 11; two-tailed *t*-test: *t*_(21)_ = 3.730, *P* = 0.0012]. **(G)** *Utx* cKO mice spent comparable time in the light box over a 5-min light-dark test [WT, *n* = 12; cKO, *n* = 11; two-tailed *t*-test: *t*_(21)_ = 0.270, *P* = 0.7845]. **(H)** *Utx* cKO mice spent significantly longer time in locating the platform during 5-day training [WT *n* = 5, cKO *n* = 10; repeated measures analysis of variance (ANOVA), followed by *Turkey post-hoc* test; Group effect: *F*_(1, 13)_ = 7.016, *P* = 0.02; Day2: *t*_(13)_ = 2.738; *P* = 0.0169; Day5: *t*_(13)_ = 2.632; *P* = 0.0207 for WT vs. cKO]. **(I)** Representative trajectory maps in the Morris water maze test. **(J)** *Utx* cKO mice showed significant decreased number of platform crossing in the Morris water maze test [WT, *n* = 5; cKO, *n* = 10; two-tailed *t*-test: *t*_(13)_ = 3.103, *P* = 0.0084]. ^*^P < 0.05; ^**^P < 0.01. Error bars, s.e.m.

To further confirm the function of *Utx* in the brain, we crossed homozygous *Utx*^f/f^ mice with transgenic *Emx1*-Cre mice, in which the endogenous *Emx1* locus directs expression of Cre recombinase specifically in the neocortex and hippocampus, to generate *Emx1-*Cre *Utx* cKO mice with *Utx* deletion in the forebrain (Figure 2A). Consistent with the observations in *Nestin-*Cre *Utx* cKO mice, these *Emx1-*Cre *Utx* cKO mice also displayed anxiety-like behaviors as indicated by decreased entry into the center zone in the open field test (Figures 2B–D), decreased entries (Figures 2E,F), and time spent (Figure 2G) in the light box in the light-dark box test. In the Morris water maze test, *Emx1-*Cre *Utx* cKO mice took prolonged time to locate the hidden platform during both training (Figure 2H) and probe trials (Figures 2I,J), indicating that impaired spatial learning and memory also exists in *Emx1-*Cre *Utx* cKO mice. These results suggest that *Utx* specific ablation in the forebrain phenocopies the behavioral deficits observed in *Nestin-*Cre *Utx*-cKO mice, further strengthening the critical role of *Utx* in regulation of cognitive behaviors.

### Down-regulation of *Utx* in adult hippocampus phenocopies the behavioral deficits as in *Utx* cKO mice

**Figure 3:**
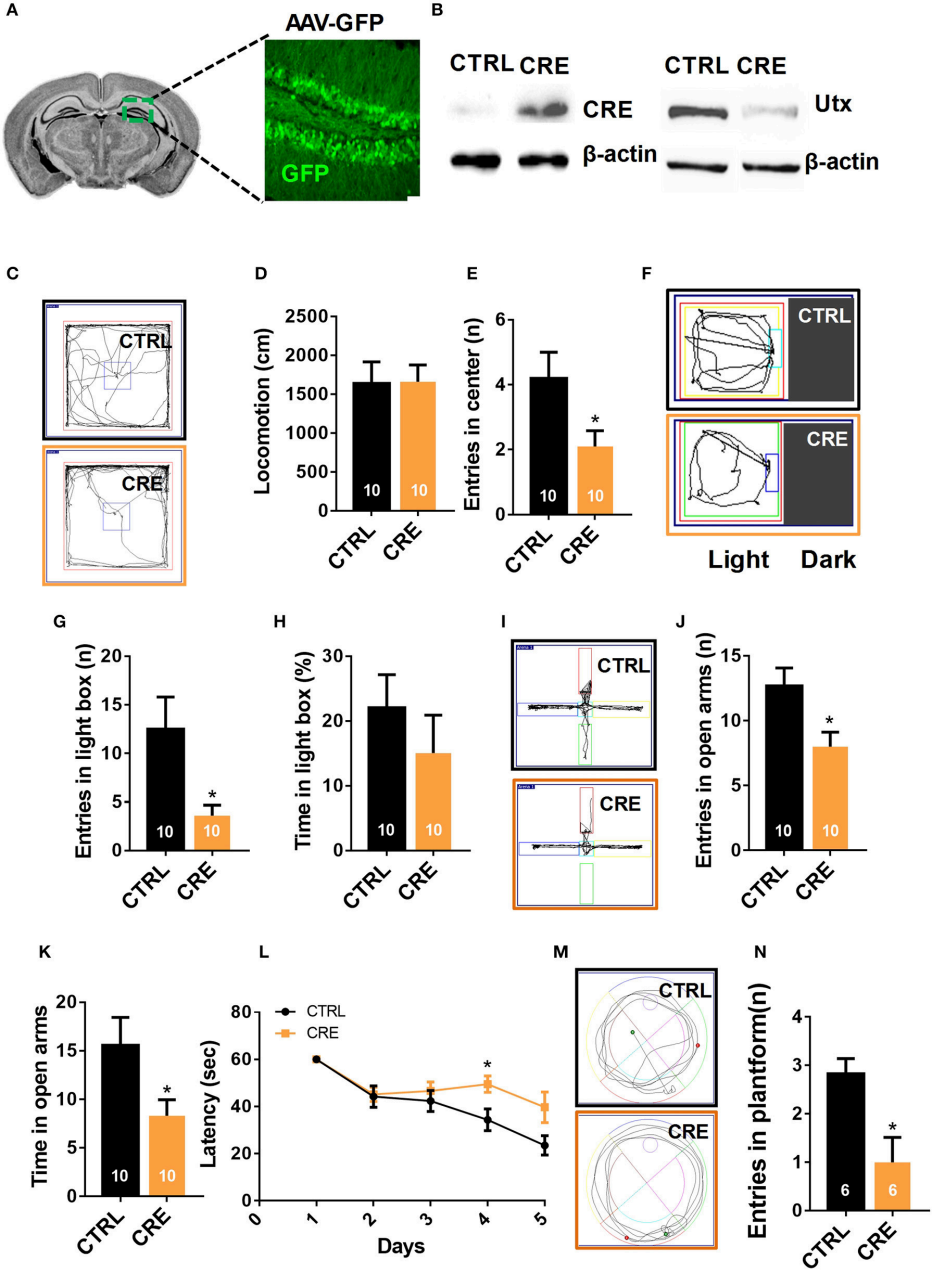
Knocking-down of *Utx* expression in adult hippocampus phenocopies the behavioral deficits observed in *Utx* cKO mice. **(A)** Schematic illustrating injection of AAV-GFP-CRE and AAV-GFP control viruses into the adult *Utx* ^f/f^ hippocampus (left), representative images showing GFP expression in the hippocampus 2 months after viral injection (right). **(B)** UTX protein level was significantly reduced in the *Utx* ^f/f^ hippocampus 2 months after AAV2/8-cre viral injection. **(C)** Representative trajectory maps of WT and *Utx* cKO mice in the open field test. **(D)** *Utx* knockdown had no effect on locomotivity [CTRL, *n* = 10; CRE, *n* = 10; two-tailed *t*-test: *t*_(18)_ = 0.05696, *P* = 0.9552]. **(E)** *Utx* knockdown resulted in less entries into the center zone during the open field test [CTRL, *n* = 10; CRE, *n* = 10; two-tailed *t*-test: *t*_(18)_ = 2.249, *P* = 0.0373]. **(F)** Representative trajectory maps of WT and *Utx* cKO mice in the light-dark field test. **(G)** Mice with *Utx* knockdown had decreased entries into the light box during 5-min light-dark box test [CTRL, *n* = 10; CRE, *n* = 10; two-tailed *t*-test: *t*_(18)_ = 2.561, *P* = 0.0196]. **(H)** Mice with *Utx* knockdown showed no significant changes of time in the light box compared to their controls over a 5-min light-dark box test [CTRL, *n* = 10; CRE, *n* = 10; two-tailed *t*-test: *t*_(18)_ = 0.9038, *P* = 0.3780]. **(I)** Representative trajectory maps of WT and *Utx* cKO mice in elevated plus maze test. **(J)** Mice with *Utx* knockdown displayed decreased entries into the open arms during 5-min elevated plus maze test [CTRL, *n* = 10; CRE, *n* = 10; two-tailed *t*-test: *t*_(18)_ = 2.655, *P* = 0.0161]. **(K)** Mice with *Utx* knockdown spent less time in the open arms over a 5-min elevated plus maze [CTRL, *n* = 10; CRE, *n* = 10; two-tailed *t*-test: *t*_(18)_ = 2.655, *P* = 0.0161]. **(L)** Mice with *Utx* knockdown spent more time in reaching the platform during 5-day training in Morris water maze test [CTRL, *n* = 7; CRE, *n* = 8; repeated measures analysis of variance (ANOVA), followed by *Turkey post-hoc* test; Group effect: *F*_(1, 13)_ = 6.038, *P* = 0.029; Day4: *t*_(13)_ = 2.663, *P* = 0.020]. **(M)** Representative trajectory maps in a water maze test. **(N)** Mice with *Utx* knockdown showed significant changes of target crossing in Morris water maze test [CTRL, *n* = 6; CRE, *n* = 6; two-tailed *t*-test: *t*_(10)_ = 3.051, *P* = 0.0122]. ^*^P < 0.05. Error bars, s.e.m.

The hippocampus is an important region in the brain in the information processing, consolidation, and storage, and responsible for cognitive ability and memory retention. It is also involved in emotional and cognitional functions (Eichenbaum, 2004). We then asked the question as to whether deletion of *Utx* in the hippocampus of adults could phenocopy the cognitive deficits exhibited in *Utx* cKO mice. To test this idea, we stereotactically injected AAV 2/8 virus expressing either GFP or Cre into the hippocampus of 8-week-old *Utx*^f/f^ mice (Figure 3A). As shown in Figure 3B, expression of *Utx* at the protein level was significantly downregulated along with Cre recombinase after virus injection. In particular, we observed that deletion of *Utx* in the hippocampus of adult *Utx*^f/f^ mice exhibited normal locomotion (Figure 3D), but showed fewer entries into the center zone (Figures 3C,E) in the open field test. This is consistent with the anxiety-like behavior and cognitional deficits in *Utx* cKO mice. Similarly, mice with *Utx* down-regulation in the hippocampus also showed decreased entries in the light chamber in the light-dark box test (Figures 3F,G,H), and fewer entries (Figures 3I,J), and time spent (Figures 3K) in the open arms in the elevated plus maze test. In the Morris water maze test, mice with *Utx* down-regulation in the hippocampus displayed the lower ability to locate the platform (Figures 3L,M,N), indicating impaired spatial learning and memory abilities. Taken together, these results indicate that deletion of *Utx* in the adult hippocampus indeed replicates the behavioral deficits observed in cKO mice in which deletion of *Utx* occurs during development, further supporting the idea that *Utx* plays critical roles in regulation of emotion and cognition in adults.

### *Utx* cKO mice display abnormalities of LTP and basal synaptic transmission in the hippocampus

**Figure 4:**
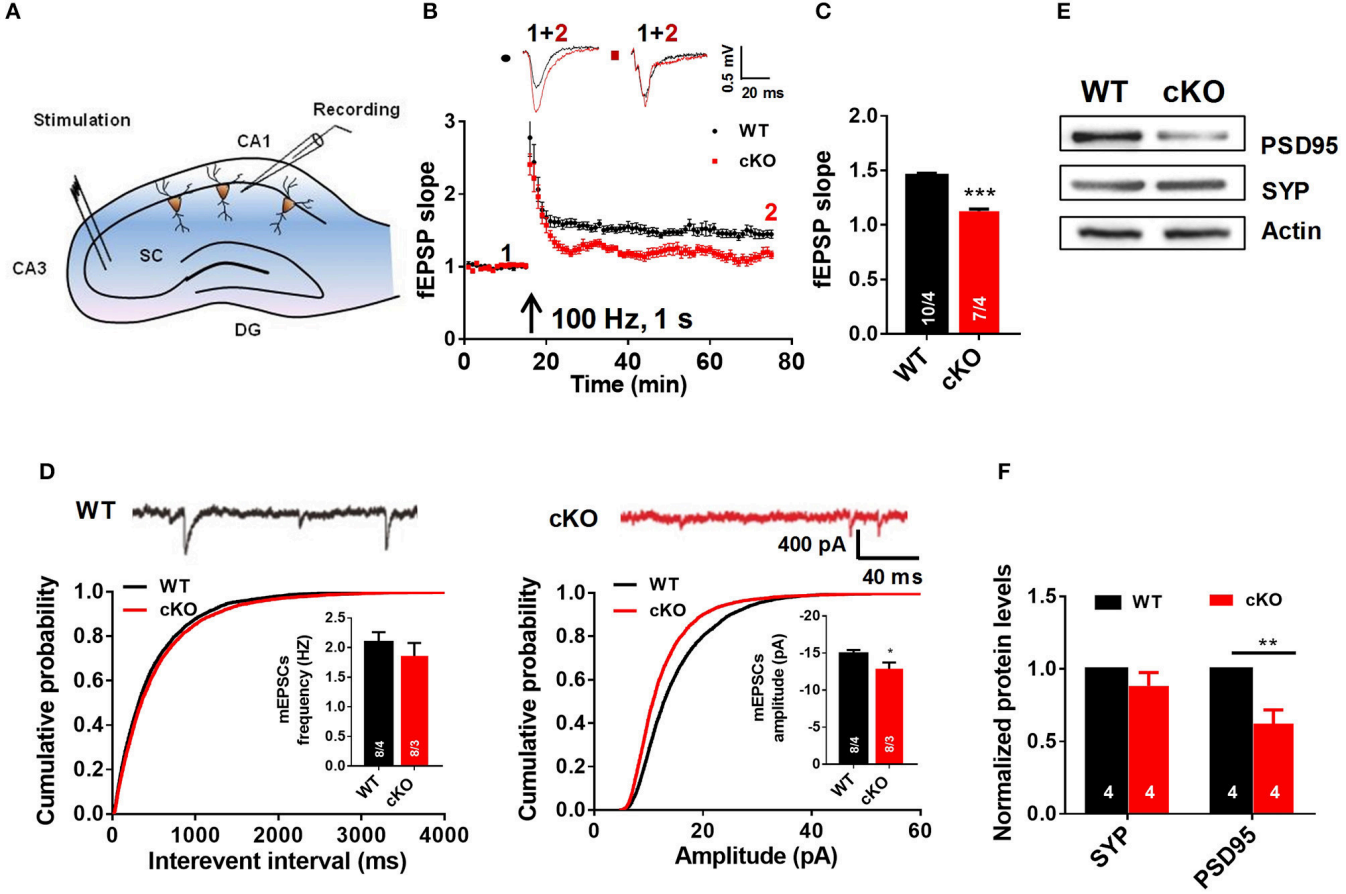
*Utx* cKO mice display abnormalities of LTP and basal synaptic transmission in CA1 of the hippocampus. **(A)** Schematic overview of the electrophysiological protocol for acute hippocampal slice recording. **(B)** Typical experiment showing time course of CA1 LTP for a single recording from WT and cKO mice. fEPSP traces before (1, black) and after (2, red) are shown in the inset above. **(C)** cKO mice showed lower LTP amplitude measured at 55–60 min post-induction [WT, 10 slices from 4 animals; cKO, 7 slices from 4 animals; two-tailed *t*-test: *t*_(338)_ = 12.07, *P* < 0.0001]. **(D)** Amplitudes [WT, 8 slices from 4 animals; cKO, 8 slices from 3 animals; two-tailed *t*-test: *t*_(14)_ = 2.211, *P* = 0.0442] but not frequencies [WT, 8 slices from 4 animals; cKO, 8 slices from 3 animals; two-tailed *t*-test: *t*_(14)_ = 0.8486, *P* = 0.4104] of spontaneous mEPSCs (monitored in 0.5 μM tetrodotoxin) was impaired in cKO CA1 neurons [top, representative traces; bottom, cumulative plots and summary graphs of the mEPSC (left) frequency and amplitude (right)]. **(E)** Representative western blotting for PSD95 and Synapsin (SYP) in total hippocampus extracts of WT and cKO mice. **(F)** Quantification of immunoreacitivity revealed a significant decrease of PSD95 expression in cKO mice. SYP protein levels were similar in cKO mice to WT mice [WT, *n* = 4; Cko, *n* = 4; two-tailed *t*-test: *t*_(6)_ = 3.951, *P* = 0.0075]. SYP protein levels were similar in cKO mice to WT mice [WT, *n* = 4; cKO, *n* = 4; two-tailed *t*-test: *t*_(6)_ = 1.262, *P* = 0.2538]. ^*^P < 0.01; ^**^P < 0.01; ^***^P < 0.001. Error bars, s.e.m.

To explore the role of *Utx* loss-of-function in hippocampal synaptic transmission and plasticity, we recorded hippocampal LTP, a cellular model for learning and memory, in acute hippocampal slices prepared from 6 to 7 week-old *Utx* cKO and WT mice. As shown in Figures 4B,C, LTP at Schaffer collateral synapses in the CA1 region (Figure 4A) was significantly impaired in cKO mice when compared with that in WT littermates. There was a significant difference in the amplitude of LTP at 55–60 min after induction between cKO and WT mice (Figures 4B,C). These results indicated that *Utx* deficiency impairs hippocampal long-term synaptic plasticity, which may underlie the behavioral deficits displayed by cKO mice.

To further determine the cause of the impaired hippocampal synaptic plasticity in *Utx* cKO mice, we recorded AMPA receptor (AMPAR)-mediated miniature excitatory postsynaptic currents (mEPSCs) in CA1 pyramidal cells using the whole-cell patchclamp technique. AMPAR mediated mEPSCs were pharmacologically isolated by bath application of the GABAA and NMDA receptor antagonists bicuculline (10 μM) and D-AP5 (25 μM) in the presence of TTX (0.5 μM). At a holding potential of −70 mV, mEPSCs displayed fast inward currents and sensitive to 20 μM DNQX, indicating AMPAR-mediated currents. As shown in Figure 4D, the amplitude, but not the frequency, of mEPSCs was significant decreased in slices from cKO mice when compared with that from WT mice. The results from electrophysiological recordings further support our speculation that *Utx* deletion impairs hippocampal synaptic transmission.

Impaired LTP and decreased amplitude of mEPSCs are most likely due to the deficits in postsynaptic function and/or reduction of functional synapses (Liao et al., 1995; Xiao et al., 2007). To test this assumption, we examined the immunoreactivity of synaptic-related proteins in the hippocampus of both WT and cKO mice. As shown in Figures 4E,F, expression of postsynaptic density protein PSD-95, a postsynaptic marker, was significantly decreased, while synapsin, a presynaptic marker, remained unchanged in cKO mice when compared to that in WT littermates. These results suggest that decreased expression of PSD-95 may lead to a deficit of postsynaptic plasticity in *Utx* cKO mice.

### Morphological abnormalities of hippocampal neurons in *Utx* cKO mice *in vivo*

**Figure 5:**
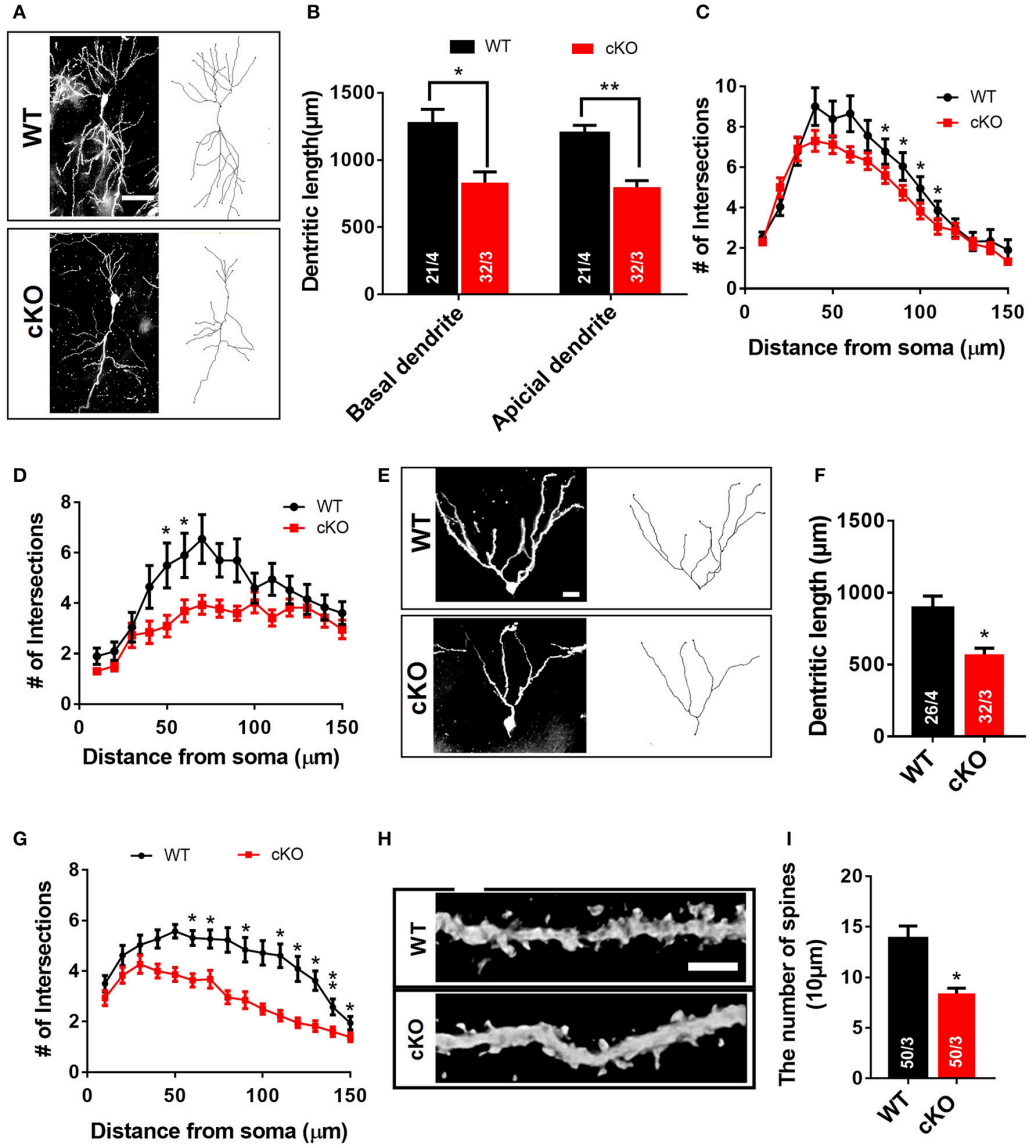
Morphological abnormalities of hippocampal neurons in *Utx* cKO mice. **(A)** Representative projection images of Golgi-stained pyramidal neurons in the hippocampal CA1 region. Scale bar, 50 μm. **(B)** Quantification of dendritic length of CA1 neurons [WT, 21 neurons from 4 mice; cKO, 32 neurons from 3 mice; two-tailed *t*-test: basal dendrite, *t*_(5)_ = 3.122, *P* = 0.0262; apical dendrite, *t*_(5)_ = 5.137, *P* = 0.0037]. **(C)** Quantification of basal dendritic number of CA1 neurons [80 μm, *t*_(5)_ = 3.090, *P* = 0.027; 90 μm, *t*_(5)_ = 2.988, *P* = 0.031; 100 μm, *t*_(5)_ = 3.313, *P* = 0.021; 110 μm, *t*_(5)_ = 2.988, *P* = 0.031]. **(D)** Reduced branching of apical dendrites was found in *Utx* deletion CA1 neurons [50 μm, *t*_(5)_ = 3.828, *P* = 0.012; 60 μm, *t*_(5)_ = 4.781, *P* = 0.005]. **(E)** Representative images of dendritic spines of Golgi-stained secondary dendrites of pyramidal neurons in the hippocampal CA1 region. Scale bars, 2 μm. **(F)** *Utx* cKO mice decreased spine density in the CA1 neurons compared to WT littermate [WT, 50 neurons in 3 mice; cKO, 50 neurons in 3 mice; two-tailed *t*-test: *t*_(4)_ = 4.082, *P* = 0.0151]. **(G)** Representative projection images of Golgi-stained neurons in the dentate gyrus. Scale bar, 50 μm. **(H)** Quantification of dendritic length of neurons in the dentate gyrus [WT, 26 neurons from 4 mice; cKO, 32 neurons from 3 mice; two-tailed *t*-test: *t*_(5)_ = 3.086, *P* = 0.0273]. **(I)** Quantification results showed that *Utx* deletion decreased dendritic branching in the dentate gyrus [60 μm, *t*_(5)_ = 3.140, *P* = 0.026; 70 μm, *t*_(5)_ = 2.869, *P* = 0.035; 90 μm, *t*_(5)_ = 3.042, *P* = 0.029; 110 μm, *t*_(5)_ = 2.587, *P* = 0.049; 120 μm, *t*_(5)_ = 3.886, *P* = 0.012; 130 μm, *t*_(5)_ = 3.054, *P* = 0.028; 140 μm, *t*_(5)_ = 6.444, *P* = 0.001; 150 μm, *t*_(5)_ = 3.095, *P* = 0.027). ^*^P < 0.05; ^**^P < 0.01. Error bars, s.e.m.

Neuronal signal integration as well as synaptic transmission and plasticity highly depend on the morphology of dendrites and their spines (Hering and Sheng, 2001). The impairment of hippocampal synaptic plasticity and the reduced PSD95 expression in *Utx* cKO mice prompted us to postulate that *Utx* deficiency may cause abnormal neuronal morphology. To determine whether disruption of *Utx* affects neuronal morphology, we traced Golgi-stained hippocampal neurons in the CA1 and dentate gyrus (DG). *Utx* cKO mice exhibited a significant reduction in dendritic length and number of branches in neurons in both the CA1 (Figures 5A–D) and DG regions (Figures 5E–G). Moreover, a reduction of spine density in hippocampal CA1 pyramidal neurons was also found in *Utx* deletion mice (Figures 5H,I). These data, together with the reduction in the postsynaptic PSD95 protein (Figure 4E), suggest an essential role for UTX in synaptic formation and dendritic development in hippocampal neurons, which in turn affect spatial learning and memory.

### *Utx* deletion in hippocampal neurons results in morphological defects *in vitro*

**Figure 6:**
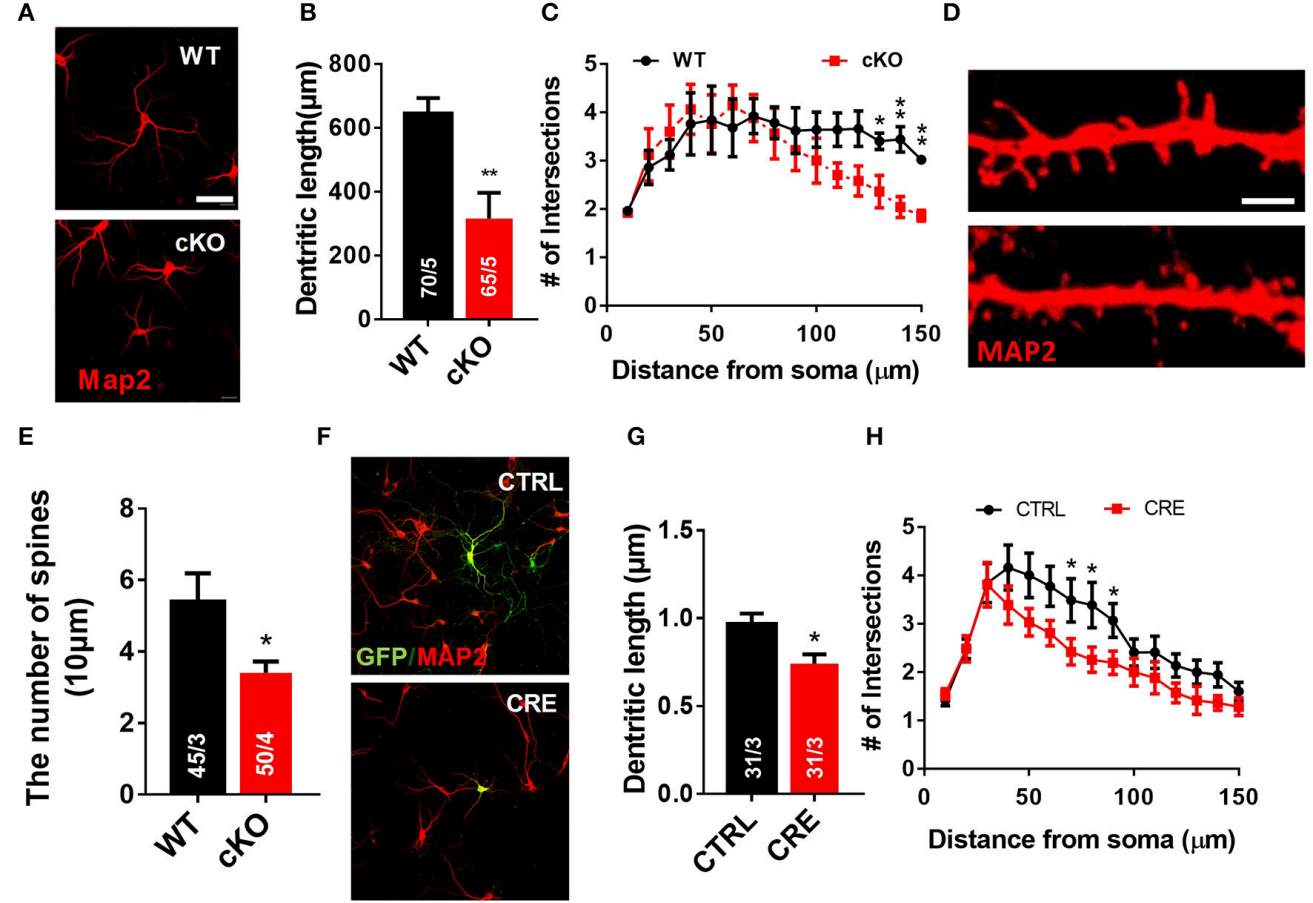
Utx deletion results in abnormal dendritic development in cultured hippocampus neurons. **(A)** Neurons from the hippocampus of *Utx* cKO and WT littermate newborn mice were cultured for 7 days *in vitro* (DIV-7), fixed and stained for expression of Map2 (red). Scale bars, 50 μm. **(B)** Quantification of dendritic length of WT and cKO P0 hippocampal neurons. **(C)** Quantification of the branching of P0 hippocampal neurons (DIV-7) [WT, 70 neurons from 5 mice; cKO, 65 neurons from 5 mice; two-tailed *t*-test: *t*_(8)_ = 3.494, *P* = 0.0082]. **(D)** Representative images of spines from Map2 stained secondary dendrites of cultured neurons (DIV-19) from P0 WT and cKO hippocampus [WT, 45 neurons from 3 mice, Cko, 50 neurons from 4 mice; two-tailed *t*-test: *t*_(8)_ = 2.309, *P* = 0.0497]. Scale bars, 2 μm. **(E)** Spine density from Map2 stained secondary dendrites of cultured neurons (DIV-19) was reduced in cKO. **(F)** Representative images of *Utx*^f/f^ hippocampal neurons infected with Cre-expressing lenti-virus (DIV7). GFP (green), Map2 (red). Scale bars, 50 μm. **(G)** Cre-mediated Utx deletion resulted in shorter dendritic length in cultured hippocampal neurons [CTRL, 31 neurons from 3 mice; CRE, 31 neurons from 3 mice; two-tailed *t*-test: *t*_(60)_ = 2.872; *P* = 0.0056]. **(H)** The dendrite complexity was decreased in cultured Utxf/f hippocampal neurons that infected with Cre-expressing lenti-virus [CTRL, 31 neurons from 3 mice; CRE, 31 neurons from 3 mice; 70 μm: *t*_(60)_ = 2.024, *P* = 0.047; 80 μm: *t*_(60)_ = 2.106, *P* = 0.039; 90 μm: *t*_(60)_ = 2.012, *P* = 0.019]. ^*^P < 0.05, ^**^P < 0.01. Error bars, s.e.m.

We next used a well-established *in vitro* primary neuron culture system to examine the effects of UTX deletion on the morphology of hippocampal neurons. Indeed, *Utx* cKO hippocampus neurons displayed a significant reduced in total dendritic length and dendritic complexity compared with control neurons isolated from littermate newborn pups (Figures 6A–C) at Day 7 *in vitro* (DIV7). At DIV19, cKO hippocampal neurons showed significant reductions in spine density (Figures 6D,E), which is consistent with the Golgi staining observations in the CA1 pyramidal neurons *in vivo* (Figures 5H,I). To further determine whether UTX regulates neuronal morphology, we acutely manipulated UTX expression by infecting in newborn hippocampus neurons isolated from *Utx*^f/f^ pups with lenti-cre viruses to delete UTX expression. As expected, we found that acute knockdown of UTX in hippocampus neurons also led to a significant decrease in total dendritic length and dendritic complexity (Figures 6F–H). These loss-of-function data in primary neurons further support our *in vivo* observation that deletion of UTX inhibits neuronal dendritic development.

### Altered expression of synaptic plasticity and cognition associated genes in the hippocampus of *Utx* cKO mice

**Figure 7:**
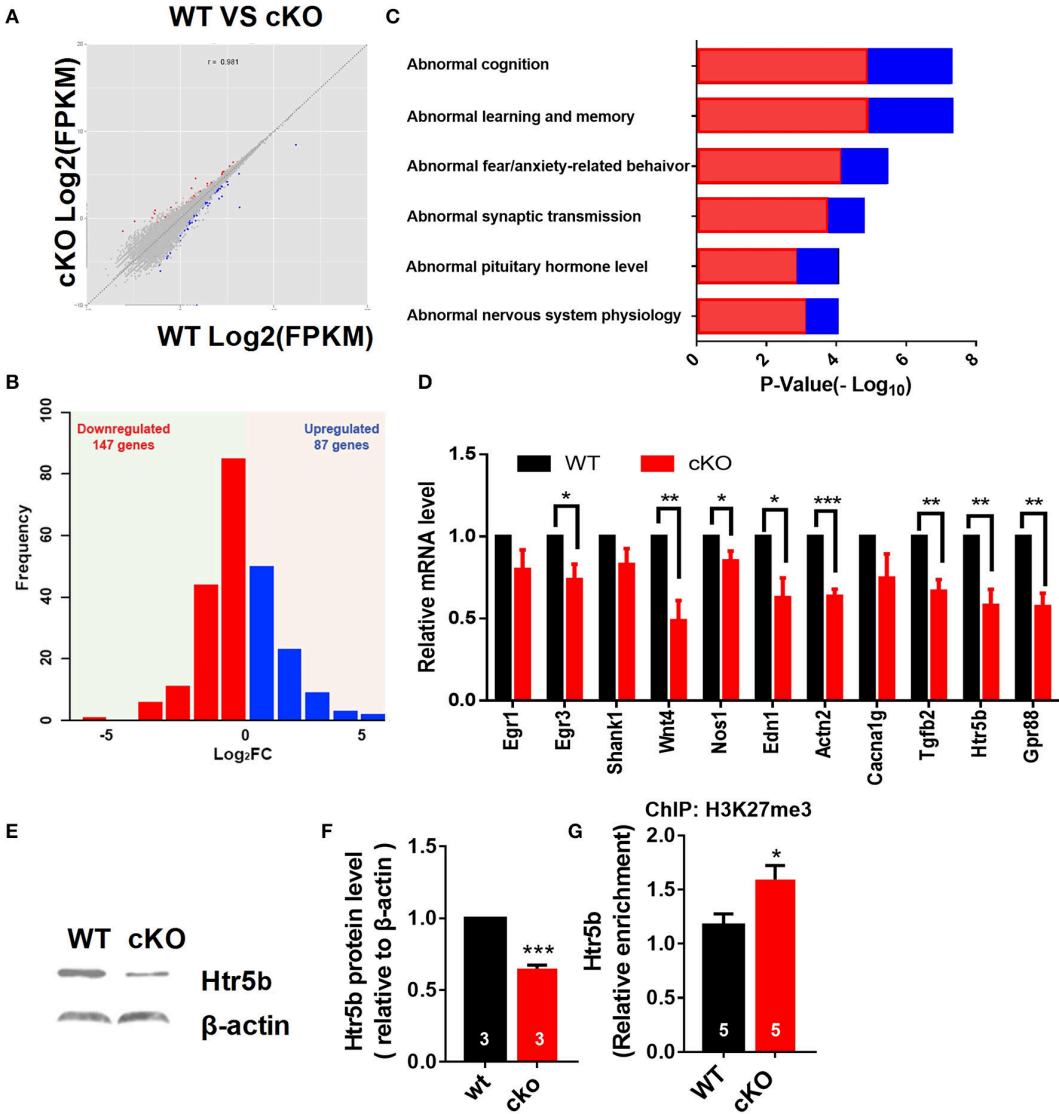
Altered expression of synaptic plasticity and cognition associated genes in the hippocampus of *Utx* cKO mice. **(A)** Correlation analysis of differential genes between cKO and WT samples. **(B)** Histograms of differentially expressed genes in the hippocampus of cKO mice compared to the littermate WT mice. **(C)** Gene ontology (GO) analysis of differentially expressed genes. Red represents down-regulated genes; Blue represents up-regulated genes. **(D)** Validation of down-regulated genes in cKO hippocampus by RT-qPCR. **(D)**, paired *t*-test; ^*^P < 0.05, ^**^P < 0.01. Error bars, s.e.m. **(E)** Representative image of western blotting analysis on *Htr5b*. **(F)** Quantification of *Htr5b* protein levels in the hippocampus of cKO and littermate WT mice [WT, *n* = 3; cKO, *n* = 3; two-tailed *t*-test: *t*_(4)_ = 14.79, *P* = 0.0001]. **(G)** ChIP assay showed that H3K27me3 was enriched at the promoter of *Htr5b* gene (*n* = 5, *P* = 0.0105). ^*^P < 0.05, ^***^P < 0.001. Error bars, s.e.m.

Next, we investigated the effects of *Utx* loss-of-function on hippocampal gene expression, in order to identify potential mechanisms that might be associated with the synaptic plasticity and cognitive dysfunction in *Utx* cKO mice. To delineate the molecular pathways regulated by *Utx*, we conducted gene expression profiling (RNA-seq) using total RNA from the hippocampus of WT and cKO mice. One hundred forty seven down-regulated and 87 up-regulated genes were identified in cKO mice when compared with WT mice (Figures 7A–C and Table 2). To uncover the genes involved in the phenotypes of *Utx* loss-of-function, we performed Gene Ontology (GO) enrichment analysis of dysregulated genes in *Utx* cKO mice and found several enriched GO terms for biological processes, including cognition, learning and memory, anxiety, and synaptic transmission (Figure 7C). We then focused on the 147 genes with decreased expression in cKO mice, as their downregulation likely resulted from a loss of *Utx*. Most of those down-regulated genes are involved in regulation of Ion transport, calcium ion binding, neurite elongation, dendritic, and synaptic formation (Table 3), suggesting an essential role of *Utx* in Cognition, Learning and Memory, Anxiety, Synaptic Formation, and Function (Figures 7C,D and Table 3).

**Table 2:** Differential gene expression in the hippocampus of *Utx* cKO mice.

**Gene ID**	**Gene name**	**cKO**	**WT**	**log_2_FC**	***P*-value**	***Q*-value**
**DOWN-REGULATED GENES**						
ENSMUSG00000092837	Rpph1	343.15316	5308.8495	−3.951475	0	0
ENSMUSG00000081824	BC002163	2.4534164	80.746173	−5.04053	1.62E-63	1.71E-59
ENSMUSG00000069919	Hba-a1	5.8219374	21.66464	−1.895771	3.20E-25	1.13E-21
ENSMUSG00000104586	4921539H07Rik	3.2608135	8.8100261	−1.433914	1.10E-24	3.32E-21
ENSMUSG00000001119	Col6a1	2.7857418	7.7819942	−1.482078	4.20E-23	1.11E-19
ENSMUSG00000069917	Hba-a2	4.8539394	17.783574	−1.873317	7.64E-19	1.62E-15
ENSMUSG00000039474	Wfs1	8.5123646	15.002664	−0.817587	8.18E-18	1.57E-14
ENSMUSG00000005672	Kit	3.5255571	6.2890673	−0.834995	9.46E-17	1.67E-13
ENSMUSG00000026504	Sdccag8	9.1889204	16.475959	−0.842395	5.64E-16	8.53E-13
ENSMUSG00000096887	Gm20594	34.423898	76.359394	−1.149395	1.13E-15	1.59E-12
ENSMUSG00000040836	Gpr161	9.6569171	15.784179	−0.708845	2.36E-15	3.12E-12
ENSMUSG00000022602	Arc	9.4851166	15.982332	−0.752741	2.56E-14	2.85E-11
ENSMUSG00000097789	Gm2115	18.661136	32.727853	−0.810482	1.92E-13	1.94E-10
ENSMUSG00000028011	Tdo2	1.2541566	3.4340393	−1.453189	8.43E-13	6.86E-10
ENSMUSG00000032327	Stra6	0.3790327	1.3524839	−1.835217	8.41E-13	6.86E-10
ENSMUSG00000038738	Shank1	7.5127037	11.280538	−0.586432	9.51E-13	7.45E-10
ENSMUSG00000039087	Rreb1	1.6818975	2.7150751	−0.690902	1.06E-12	7.99E-10
ENSMUSG00000029361	Nos1	3.95298	6.2713571	−0.665837	1.67E-12	1.18E-09
ENSMUSG00000028909	Ptpru	3.1446904	5.6113616	−0.835433	2.75E-12	1.87E-09
ENSMUSG00000020866	Cacna1g	2.4304661	4.0903474	−0.75099	3.01E-12	1.99E-09
ENSMUSG00000062372	Otof	0.1484716	0.7338461	−2.305291	1.40E-11	7.23E-09
ENSMUSG00000037428	Vgf	5.4764434	9.2877787	−0.762094	1.68E-11	8.34E-09
ENSMUSG00000022416	Cacna1i	4.5026666	7.0137187	−0.6394	4.79E-11	2.30E-08
ENSMUSG00000021803	Cdhr1	0.7274866	2.4312039	−1.740678	7.98E-11	3.59E-08
ENSMUSG00000037138	Aff3	4.9159226	7.7216196	−0.651441	1.48E-10	6.22E-08
ENSMUSG00000030592	Ryr1	1.8583275	3.1236378	−0.749222	1.61E-10	6.57E-08
ENSMUSG00000027520	Zdbf2	1.0423908	1.7822945	−0.773839	2.32E-10	8.46E-08
ENSMUSG00000033377	Palmd	2.1376507	3.8711601	−0.85674	2.92E-10	1.03E-07
ENSMUSG00000036943	Rab8b	10.890041	16.662915	−0.613631	3.76E-10	1.31E-07
ENSMUSG00000037579	Kcnh3	13.105166	20.217393	−0.625461	5.24E-10	1.79E-07
ENSMUSG00000032500	Dclk3	4.9807473	9.4493604	−0.923854	5.73E-10	1.92E-07
ENSMUSG00000037369	Kdm6a	3.9870515	6.2300865	−0.64393	2.95E-09	9.17E-07
ENSMUSG00000102543	Pcdhgc5	5.3198372	8.0448876	−0.59669	4.11E-09	1.21E-06
ENSMUSG00000036882	Arhgap33	4.5941122	7.9284449	−0.787252	7.84E-09	2.17E-06
ENSMUSG00000039239	Tgfb2	3.4664434	5.4757102	−0.65959	8.32E-09	2.23E-06
ENSMUSG00000038418	Egr1	2.6017263	4.4423229	−0.771845	1.52E-08	3.75E-06
ENSMUSG00000079110	Capn3	1.3940483	2.7705691	−0.990902	1.51E-08	3.75E-06
ENSMUSG00000030703	Gdpd3	0.4099224	1.7892865	−2.125962	1.60E-08	3.90E-06
ENSMUSG00000036777	Anln	2.4000525	4.4271812	−0.883322	2.75E-08	6.43E-06
ENSMUSG00000041577	Prelp	1.124159	2.713794	−1.271465	4.08E-08	9.09E-06
ENSMUSG00000032289	Thsd4	2.2038886	3.3192968	−0.590826	4.26E-08	9.30E-06
ENSMUSG00000037843	Vstm2l	10.550429	18.898041	−0.840935	4.52E-08	9.77E-06
ENSMUSG00000023886	Smoc2	10.293695	16.079821	−0.64349	4.88E-08	1.04E-05
ENSMUSG00000021867	Tmem254b	0.4440248	1.7208204	−1.954384	9.93E-08	2.00E-05
ENSMUSG00000081738	Hmgb1-ps2	0	3.4556435	#NAME?	1.37E-07	2.58E-05
ENSMUSG00000052374	Actn2	1.2598146	2.7926344	−1.148415	2.28E-07	4.03E-05
ENSMUSG00000044393	Dsg2	0.4516196	1.1284631	−1.321179	2.90E-07	4.94E-05
ENSMUSG00000052861	Dnah6	0.5956064	1.1700731	−0.974167	3.15E-07	5.25E-05
ENSMUSG00000043439	E130012A19Rik	2.2828718	5.1288012	−1.167772	3.32E-07	5.45E-05
ENSMUSG00000049892	Rasd1	3.400498	7.5305922	−1.147017	3.40E-07	5.54E-05
ENSMUSG00000020635	Fkbp1b	12.590353	22.269622	−0.822758	5.83E-07	8.92E-05
ENSMUSG00000044783	Hjurp	1.8172029	2.8328347	−0.640527	5.79E-07	8.92E-05
ENSMUSG00000103004	2900002M20Rik	14.525397	33.299838	−1.196938	6.68E-07	0.0001009
ENSMUSG00000052305	Hbb-bs	9.7599188	15.919328	−0.705838	1.25E-06	0.0001663
ENSMUSG00000031785	Adgrg1	6.6330473	9.9878521	−0.590503	1.98E-06	0.0002445
ENSMUSG00000020096	Tbata	1.2678716	2.5992368	−1.035679	2.19E-06	0.0002674
ENSMUSG00000041798	Gck	0.2917662	1.0675203	−1.871379	2.42E-06	0.0002882
ENSMUSG00000030889	Vwa3a	1.5389782	2.6433614	−0.780401	3.24E-06	0.0003721
ENSMUSG00000000805	Car4	7.1546275	11.026463	−0.624022	3.42E-06	0.000386
ENSMUSG00000024998	Plce1	0.7324494	1.1872262	−0.696794	3.41E-06	0.000386
ENSMUSG00000021260	Hhipl1	0.6759403	2.1260714	−1.653222	4.17E-06	0.0004511
ENSMUSG00000073125	Xlr3b	0.0417671	0.3628541	−3.118952	5.40E-06	0.0005611
ENSMUSG00000102465	Gm38198	5.381579	8.3222294	−0.628941	5.89E-06	0.0005935
ENSMUSG00000019817	Plagl1	0.5085594	0.9412472	−0.888157	6.22E-06	0.0006206
ENSMUSG00000078606	Gm4070	0.8009457	1.4839985	−0.889713	6.81E-06	0.0006699
ENSMUSG00000073418	C4b	2.345801	3.7833854	−0.689597	7.94E-06	0.0007536
ENSMUSG00000033730	Egr3	7.7375739	13.22392	−0.773197	9.65E-06	0.0008705
ENSMUSG00000036357	Gpr101	1.6997205	3.0062153	−0.822651	9.67E-06	0.0008705
ENSMUSG00000045868	Gvin1	0.957694	1.7212828	−0.845848	9.86E-06	0.0008831
ENSMUSG00000026072	Il1r1	1.4651284	2.3646069	−0.690573	1.08E-05	0.0009446
ENSMUSG00000032572	Col6a4	0.0624639	0.3461836	−2.470443	1.10E-05	0.0009549
ENSMUSG00000072676	Tmem254a	0.5550245	1.596114	−1.52394	1.12E-05	0.0009697
ENSMUSG00000046215	Rprml	19.080628	29.071677	−0.607506	1.30E-05	0.0011094
ENSMUSG00000030877	4933427G17Rik	0.1766952	1.0163118	−2.524008	1.52E-05	0.0012533
ENSMUSG00000075270	Pde11a	1.7476556	2.903545	−0.732395	1.59E-05	0.001295
ENSMUSG00000038793	Lefty1	1.8650257	4.0304326	−1.111739	1.84E-05	0.0014521
ENSMUSG00000050271	D8Ertd82e	1.0316876	1.6804305	−0.703825	2.06E-05	0.0015863
ENSMUSG00000049577	Zfpm1	3.708777	5.7812023	−0.640426	2.08E-05	0.0015955
ENSMUSG00000097141	Gm10524	3.5140602	6.6409369	−0.918248	2.16E-05	0.0016369
ENSMUSG00000072680	Tmem254c	0.6184067	1.9103469	−1.627207	2.62E-05	0.0019264
ENSMUSG00000074934	Grem1	1.0848422	2.2677239	−1.06376	2.80E-05	0.0020321
ENSMUSG00000022018	Rgcc	12.574418	20.589241	−0.711399	3.26E-05	0.0022636
ENSMUSG00000036856	Wnt4	1.0262968	2.1590117	−1.072923	3.82E-05	0.0025544
ENSMUSG00000050074	Spink8	3.1122839	6.547285	−1.072923	3.82E-05	0.0025544
ENSMUSG00000039706	Ldb2	1.9941852	3.2031902	−0.68371	4.12E-05	0.0027233
ENSMUSG00000078137	Ankrd63	1.5294515	2.7127453	−0.826739	5.82E-05	0.0036436
ENSMUSG00000005958	Ephb3	1.4756735	2.2838844	−0.630116	6.12E-05	0.0037987
ENSMUSG00000078922	Tgtp1	0.1026696	0.6228395	−2.600852	6.68E-05	0.0040471
ENSMUSG00000032925	Itgbl1	0.967797	1.7808684	−0.879805	6.80E-05	0.0040878
ENSMUSG00000051435	Fhad1	0.4853785	1.0374421	−1.095849	7.30E-05	0.004351
ENSMUSG00000075284	Wipf1	0.5486925	1.0620244	−0.952747	7.77E-05	0.0045929
ENSMUSG00000095562	Gm21887	2.5026425	5.490305	−1.133434	8.58E-05	0.0049442
ENSMUSG00000079022	Col22a1	0.3530049	0.7650955	−1.115952	0.000117	0.0064454
ENSMUSG00000001870	Ltbp1	0.5714603	0.9680695	−0.760457	0.0001194	0.0065125
ENSMUSG00000028172	Tacr3	0.2080382	0.8412479	−2.015682	0.0001248	0.0067355
ENSMUSG00000102407	Gm37862	4.6344067	7.2189348	−0.639401	0.0001657	0.0086999
ENSMUSG00000053128	Rnf26	1.2120026	1.8837514	−0.636216	0.0001842	0.0094599
ENSMUSG00000086902	Gm11728	0.8433509	3.1156524	−1.885329	0.0001841	0.0094599
ENSMUSG00000050511	Oprd1	2.724796	4.3070121	−0.660539	0.000186	0.0095277
ENSMUSG00000090066	1110002E22Rik	0.3834034	0.775228	−1.015758	0.000199	0.0100245
ENSMUSG00000002944	Cd36	0.0234352	0.2106132	−3.167848	0.0002368	0.0114672
ENSMUSG00000102386	2900022M07Rik	11.061809	17.201915	−0.636982	0.000263	0.0124171
ENSMUSG00000026890	Lhx6	1.2525381	1.9723975	−0.655096	0.0002755	0.0128412
ENSMUSG00000005716	Pvalb	7.1324289	10.943292	−0.617581	0.0002766	0.0128601
ENSMUSG00000078235	Fam43b	3.7732819	6.0964171	−0.692142	0.0002862	0.0132083
ENSMUSG00000029096	Htra3	0.0149068	0.2277448	−3.933375	0.0002895	0.0132559
ENSMUSG00000032269	Htr3a	3.0070845	5.2358214	−0.80005	0.0003181	0.0142605
ENSMUSG00000050534	Htr5b	1.2638996	2.9076496	−1.201972	0.0003571	0.015611
ENSMUSG00000001103	Sebox	0.825012	1.9771303	−1.260921	0.0004011	0.0170936
ENSMUSG00000036446	Lum	1.1786245	2.6267616	−1.156181	0.0004193	0.0177444
ENSMUSG00000075316	Scn9a	0.3132207	0.5406202	−0.787436	0.0004671	0.0192225
ENSMUSG00000053093	Myh7	0.2539611	0.5751211	−1.179258	0.0004948	0.0201215
ENSMUSG00000085565	Gm15721	0	0.3189092	#NAME?	0.0005046	0.0203497
ENSMUSG00000049493	Pls1	1.399529	2.4514467	−0.808692	0.0005387	0.0213035
ENSMUSG00000005397	Nid1	1.553279	2.4427858	−0.65321	0.000576	0.0226093
ENSMUSG00000021367	Edn1	0.2503885	1.0126457	−2.015889	0.0005889	0.0229472
ENSMUSG00000060402	Chst8	0.7692233	1.2923727	−0.748548	0.0006317	0.0243015
ENSMUSG00000039059	Hrh3	1.2506494	1.916005	−0.615424	0.0006386	0.0245201
ENSMUSG00000036915	Kirrel2	0.360044	0.7909152	−1.13535	0.0006737	0.0254718
ENSMUSG00000055972	2810407A14Rik	0.0505391	0.4087904	−3.015889	0.0007681	0.0286118
ENSMUSG00000021798	Ldb3	0.2229812	0.61547	−1.464766	0.0008079	0.0294995
ENSMUSG00000012296	Tjap1	3.2044151	5.0475271	−0.655516	0.0008417	0.030237
ENSMUSG00000050022	Amz1	0.6373296	1.0685479	−0.74554	0.0008496	0.0304674
ENSMUSG00000023918	Adgrf4	0.3287518	0.8493919	−1.369431	0.0008714	0.0310926
ENSMUSG00000031549	Ido2	0.0470734	0.2820339	−2.582886	0.0009088	0.0318357
ENSMUSG00000040852	Plekhh2	1.235951	1.9532955	−0.660289	0.0009138	0.0319563
ENSMUSG00000100303	2600014E21Rik	0.0949507	0.2986401	−1.653158	0.0009368	0.0325985
ENSMUSG00000024565	Sall3	0.3409566	0.7404627	−1.118839	0.0009501	0.0330088
ENSMUSG00000048200	Cracr2b	0.1215388	0.4733226	−1.961407	0.0009573	0.0330959
ENSMUSG00000001506	Col1a1	0.7759423	1.3439044	−0.792409	0.0009987	0.0343012
ENSMUSG00000086873	Gm15672	0	0.7141694	#NAME?	0.0010036	0.0344168
ENSMUSG00000028885	Smpdl3b	0.73149	1.9282666	−1.398394	0.0010158	0.0347666
ENSMUSG00000042425	Frmpd3	1.1972007	2.1756713	−0.861796	0.0010215	0.0348465
ENSMUSG00000033880	Lgals3bp	0.7918202	1.5451558	−0.964508	0.0010524	0.0354552
ENSMUSG00000032109	Nlrx1	1.4814639	2.4908971	−0.749642	0.0010762	0.0359733
ENSMUSG00000041633	Kctd12b	0.8244747	1.4990826	−0.862533	0.0010905	0.0362858
ENSMUSG00000047419	Cmya5	0.8915644	1.3427781	−0.59081	0.0011048	0.0366969
ENSMUSG00000021943	Gdf10	1.7559262	3.2260986	−0.877558	0.0011217	0.036981
ENSMUSG00000040856	Dlk1	0.0466162	0.1623244	−1.799976	0.0011848	0.038389
ENSMUSG00000074873	AI606181	1.4694749	2.6725563	−0.86292	0.0012829	0.0405048
ENSMUSG00000054453	Sytl5	2.8484561	4.6993022	−0.722266	0.001308	0.0409845
ENSMUSG00000107115	Gm43052	1.4421544	3.0779731	−1.093755	0.0013108	0.0409845
ENSMUSG00000054072	Iigp1	0.62687	1.4365474	−1.196367	0.0013235	0.0411792
ENSMUSG00000000739	Sult5a1	0.0636011	0.4858457	−2.933375	0.0013738	0.0422495
ENSMUSG00000021469	Msx2	0.0486417	0.3715717	−2.933375	0.0013738	0.0422495
ENSMUSG00000042489	Clspn	0.1287045	0.3566211	−1.47033	0.0015953	0.0479824
ENSMUSG00000104860	Gm42510	0.1732834	0.817611	−2.238281	0.0016407	0.0489624
**UP-REGULATED GENES**						
ENSMUSG00000038155	Gstp2	23.757467	3.0826513	2.9461374	8.39E-31	5.91E-27
ENSMUSG00000097451	Rian	40.347123	23.087851	0.8053319	8.45E-30	4.47E-26
ENSMUSG00000058126	Tpm3-rs7	11.240998	2.2541321	2.3181261	1.47E-29	6.23E-26
ENSMUSG00000102657	Gm37899	6.0712625	2.6826188	1.1783545	1.27E-20	2.98E-17
ENSMUSG00000059898	Dsc3	1.4567613	0.1491449	3.2879778	5.06E-16	8.24E-13
ENSMUSG00000070695	Cntnap5a	16.955086	10.682688	0.6664434	6.02E-15	7.49E-12
ENSMUSG00000036907	C1ql2	62.412139	37.84579	0.7216938	1.26E-14	1.48E-11
ENSMUSG00000071793	2610005L07Rik	17.288537	10.092946	0.7764684	3.46E-13	3.33E-10
ENSMUSG00000045573	Penk	15.77819	7.5352583	1.0662028	4.73E-13	4.36E-10
ENSMUSG00000058626	Capn11	1.9167958	0.1538104	3.6394717	5.20E-13	4.58E-10
ENSMUSG00000044071	Fam19a2	34.610743	22.380722	0.6289633	1.62E-12	1.18E-09
ENSMUSG00000052942	Glis3	5.4254751	3.1556836	0.7817969	3.14E-12	2.01E-09
ENSMUSG00000060803	Gstp1	87.486572	50.865489	0.7823745	4.10E-12	2.48E-09
ENSMUSG00000053819	Camk2d	1.4764576	0.8663897	0.7690518	4.91E-12	2.88E-09
ENSMUSG00000029311	Hsd17b11	7.985139	3.8603376	1.0485905	1.69E-11	8.34E-09
ENSMUSG00000062151	Unc13c	2.7838588	1.4295702	0.9615046	6.27E-11	2.95E-08
ENSMUSG00000026278	Bok	13.041501	7.4492831	0.8079364	2.15E-10	8.12E-08
ENSMUSG00000048047	Zbtb33	3.6364606	1.9482001	0.9003931	2.74E-10	9.81E-08
ENSMUSG00000051985	Igfn1	0.4683386	0.0701504	2.7390283	6.59E-09	1.86E-06
ENSMUSG00000024211	Grm8	1.1899464	0.5102175	1.2217123	1.47E-08	3.70E-06
ENSMUSG00000097312	Gm26870	1.0391809	0.1262102	3.0415465	7.45E-08	1.53E-05
ENSMUSG00000042567	Nek10	3.4050952	1.7816123	0.9345117	8.62E-08	1.75E-05
ENSMUSG00000019997	Ctgf	2.0980084	0.7763353	1.4342687	1.23E-07	2.41E-05
ENSMUSG00000021303	Gng4	8.1073368	4.5000252	0.849295	1.26E-07	2.45E-05
ENSMUSG00000035131	Brinp3	2.8082278	1.6059587	0.8062252	1.74E-07	3.18E-05
ENSMUSG00000063954	Hist2h2aa2	63.734222	38.128119	0.7412129	2.39E-07	4.18E-05
ENSMUSG00000097230	Gm26853	15.246984	10.150365	0.5869923	2.89E-07	4.94E-05
ENSMUSG00000096995	2810029C07Rik	8.7791467	4.3730355	1.0054457	3.53E-07	5.70E-05
ENSMUSG00000028648	Ndufs5	39.922194	26.017343	0.6177174	3.69E-07	5.83E-05
ENSMUSG00000059203	I11rap12	1.2364772	0.4273698	1.5326789	7.35E-07	0.0001066
ENSMUSG00000051136	Ghsr	1.6991839	0.5949242	1.5140643	1.07E-06	0.0001478
ENSMUSG00000103914	Gm38073	1.1744307	0.2638432	2.1542089	1.70E-06	0.0002166
ENSMUSG00000040569	Slc26a7	0.3654985	0.0142764	4.6781573	1.76E-06	0.0002218
ENSMUSG00000108088	RP24-385J11.1	14.907757	9.5440336	0.6433922	2.85E-06	0.0003336
ENSMUSG00000078958	Atp6ap1l	0.8108886	0.0331145	4.6139705	3.34E-06	0.0003822
ENSMUSG00000040473	Cfap69	1.7048071	0.9877702	0.7873611	4.14E-06	0.000451
ENSMUSG00000046818	Ddit4l	3.802808	1.8567163	1.0343117	5.84E-06	0.0005935
ENSMUSG00000029622	Arpc1b	1.7264657	0.8850166	0.9640452	8.07E-06	0.0007618
ENSMUSG00000104343	5730408A14Rik	7.8302722	4.0485645	0.951652	8.58E-06	0.0008032
ENSMUSG00000034295	Fhod3	3.7263115	2.1945351	0.7638329	1.59E-05	0.0012951
ENSMUSG00000032323	Cyp11a1	1.1502956	0.4494374	1.3558125	2.00E-05	0.0015485
ENSMUSG00000015217	Hmgb3	4.7493582	2.6051959	0.8663407	2.52E-05	0.0018551
ENSMUSG00000029335	Bmp3	1.1143181	0.5789269	0.944708	2.90E-05	0.0020905
ENSMUSG00000038242	Aox4	0.4190221	0.1062038	1.9801914	2.96E-05	0.0021251
ENSMUSG00000031297	Slc7a3	1.9681051	1.014805	0.9556046	3.18E-05	0.0022151
ENSMUSG00000102760	Gm37258	5.17083	2.2693611	1.1881097	3.38E-05	0.0023297
ENSMUSG00000028883	Sema3a	0.6547767	0.3369588	0.9584308	3.87E-05	0.0025809
ENSMUSG00000010122	Slc47a1	0.7393762	0.1845685	2.0021525	4.90E-05	0.0031498
ENSMUSG00000096401	Gm21811	32.713415	21.552431	0.6020317	4.94E-05	0.0031551
ENSMUSG00000006931	P3h4	5.4143505	2.9267072	0.8875099	6.56E-05	0.0040147
ENSMUSG00000029843	Slc13a4	2.009392	1.0649389	0.9159884	7.91E-05	0.0046565
ENSMUSG00000098900	Gm18190	3.4097591	0.7396174	2.2048188	7.99E-05	0.004663
ENSMUSG00000031654	Cbln1	1.0749988	0.3370022	1.673505	8.23E-05	0.0047601
ENSMUSG00000029687	Ezh2	2.2360547	1.3881729	0.6877682	9.58E-05	0.0054187
ENSMUSG00000030092	Cntn6	1.452663	0.7853919	0.8872153	9.93E-05	0.0055888
ENSMUSG00000063087	Gm10125	1.7129088	0.976673	0.8105008	0.0001025	0.0057534
ENSMUSG00000089714	Gm16092	1.3829571	0.36553	1.9196948	0.0001248	0.0067355
ENSMUSG00000095730	Vmn2r29	2.8288498	1.2710985	1.1541398	0.000137	0.0073028
ENSMUSG00000038252	Ncapd2	2.8972102	1.903396	0.6060886	0.0001432	0.0076141
ENSMUSG00000047904	Sstr2	3.957747	2.476635	0.6762981	0.0001986	0.0100245
ENSMUSG00000035258	Abi3bp	0.5578211	0.1113993	2.3240617	0.0002191	0.0107574
ENSMUSG00000060678	Hist1h4c	41.358725	25.371849	0.704963	0.0002717	0.0127098
ENSMUSG00000038775	Vill	0.3915239	0.1005329	1.9614329	0.0003511	0.0154443
ENSMUSG00000046719	Nxph3	4.8935604	2.6385975	0.8911132	0.0003569	0.015611
ENSMUSG00000107567	RP23-279L13.2	2.4131539	0.5004515	2.2696178	0.0003602	0.015714
ENSMUSG00000084799	Ino80dos	2.7823365	1.5710203	0.8245951	0.0004015	0.0170936
ENSMUSG00000043773	1700048O20Rik	3.6341623	1.6061503	1.178016	0.0004207	0.0177488
ENSMUSG00000036412	Arsi	1.085107	0.3250371	1.7391611	0.0004461	0.0185056
ENSMUSG00000028970	Abcb1b	1.0376385	0.5683707	0.8683997	0.0004955	0.0201215
ENSMUSG00000090272	Mndal	2.0502162	1.1230144	0.8683997	0.0004955	0.0201215
ENSMUSG00000032291	Crabp1	1.4637327	0	Inf	0.0005046	0.0203497
ENSMUSG00000062393	Dgkk	0.1068457	0	Inf	0.0005046	0.0203497
ENSMUSG00000032387	Rbpms2	0.8481075	0.2365363	1.8421857	0.0005304	0.0210926
ENSMUSG00000038257	Glra3	1.0495858	0.4366758	1.2651856	0.0005788	0.0226653
ENSMUSG00000103313	Gm38357	3.2555465	1.6412155	0.9881351	0.0005795	0.0226653
ENSMUSG00000068696	Gpr88	0.5517432	0.1077955	2.3556999	0.0005953	0.0231542
ENSMUSG00000068962	Zfp114	1.1374987	0.5943184	0.936557	0.0006082	0.0235238
ENSMUSG00000042268	Slc26a9	0.4867703	0.1458118	1.7391332	0.0007638	0.0285014
ENSMUSG00000027932	Slc27a3	2.1175637	1.2478491	0.7629619	0.0007765	0.0288737
ENSMUSG00000059852	Kcng2	5.381946	3.579995	0.5881703	0.0008809	0.0313238
ENSMUSG00000073164	2410018L13Rik	0.9635881	0.4768918	1.0147546	0.0009363	0.0325985
ENSMUSG00000053714	4732471J01Rik	2.9049679	1.8600372	0.6431907	0.0009946	0.0342163
ENSMUSG00000027792	Bche	1.2099084	0.6752757	0.8413494	0.001076	0.0359733
ENSMUSG00000016918	Sulf1	0.611746	0.3483563	0.8123692	0.0012599	0.0400929
ENSMUSG00000045996	Polr2k	15.618181	9.8627626	0.6631627	0.0013219	0.0411792
ENSMUSG00000074283	Zfp109	1.9004964	1.1603279	0.7118437	0.0013482	0.0417029
ENSMUSG00000073542	Cep76	3.0731451	1.8779762	0.7105371	0.0015965	0.0479824

**Table 3:** Gene Ontology analysis of down-regulated genes under *Utx* conditional knockout.

**ID**	**Name**	***p*-Value**	**FDR B&H**	**FDR B&Y**	**Bonferroni**	**Number of genes from input**	**Number of genes in annotation**
GO:0007268	Synaptic transmission	1.14E−06	2.95E−04	2.61E−03	4.43E−03	21	680
GO:0043269	Regulation of ion transport	3.42E−06	5.16E−04	4.57E−03	1.33E−02	20	669
GO:0006811	Ion transport	4.10E−06	5.69E−04	5.03E−03	1.59E−02	34	1627
GO:0005509	Calcium ion binding	9.15E−05	2.67E−02	1.92E−01	6.76E−02	18	710
GO:0097458	Neuron part	1.93E−05	1.04E−03	6.80E−03	7.31E−03	31	1552
GO:0043005	Neuron projection	6.97E−05	1.65E−03	1.08E−02	2.64E−02	25	1199
GO:0044456	Synapse part	7.18E−04	1.01E−02	6.57E−02	2.72E−01	16	714
GO:0045202	Synapse	9.14E−04	1.16E−02	7.53E−02	3.47E−01	18	876
GO:0043025	Neuronal cell body	1.44E−03	1.65E−02	1.08E−01	5.45E−01	14	620
GO:0097060	Synaptic membrane	1.59E−03	1.72E−02	1.12E−01	6.01E−01	9	300
GO:0030425	Dendrite	2.70E−03	2.43E−02	1.59E−01	1.00E+00	13	592

We then further examined some of those down-regulated genes relevant for synaptic plasticity and cognition found to be altered by RNA-seq. Using RT-qPCR, we verified that several genes critical for synaptic plasticity, and/or dendrite development including nitric oxide synthase 1 (*Nos1*), actin filament cross-linker α-Actinin-2 (*Actn2*), zinc finger transcription factor *Egr3*, transforming growth factor-β2 (*Tgfb2*), and *Wnt4* were down-regulated in the hippocampus of *Utx* cKO mice (Figure 7D). Several other essential genes (Gpr88 and Edn1) involved in mental disorder, learning, and memory were also suppressed in the hippocampus of *Utx* cKO mice (Figure 7D). Amongst the down-regulated genes, we were particularly interested in *Htr5b*, a functionally unknown serotonin receptor. Serotonin and its receptors have been implicated in dendrite morphology, synaptic transmission and cognition (Wirth et al., 2016). To validate the results from RNA-seq analysis, we conducted qPCR and western blotting analyses and observed that *Utx* deletion significantly decreased expression of *Htr5b* in the hippocampus of cKO mice (Figures 7D–F). To further verify whether H3K27me3 enriches on the promoter of *Htr5b*, we performed ChIP analysis and found that H3K27me3 was enriched in the promoter region of *Htr5b* (Figure 7G), suggesting that *Utx* mediated H3K27me3 demethylase activity is required for the alteration in *Htr5b* in the hippocampus.

### Htr5b modulates neuronal morphology, and its gain-of-function rescues the impairment of neuronal morphology in *Utx* cKO neurons

**Figure 8:**
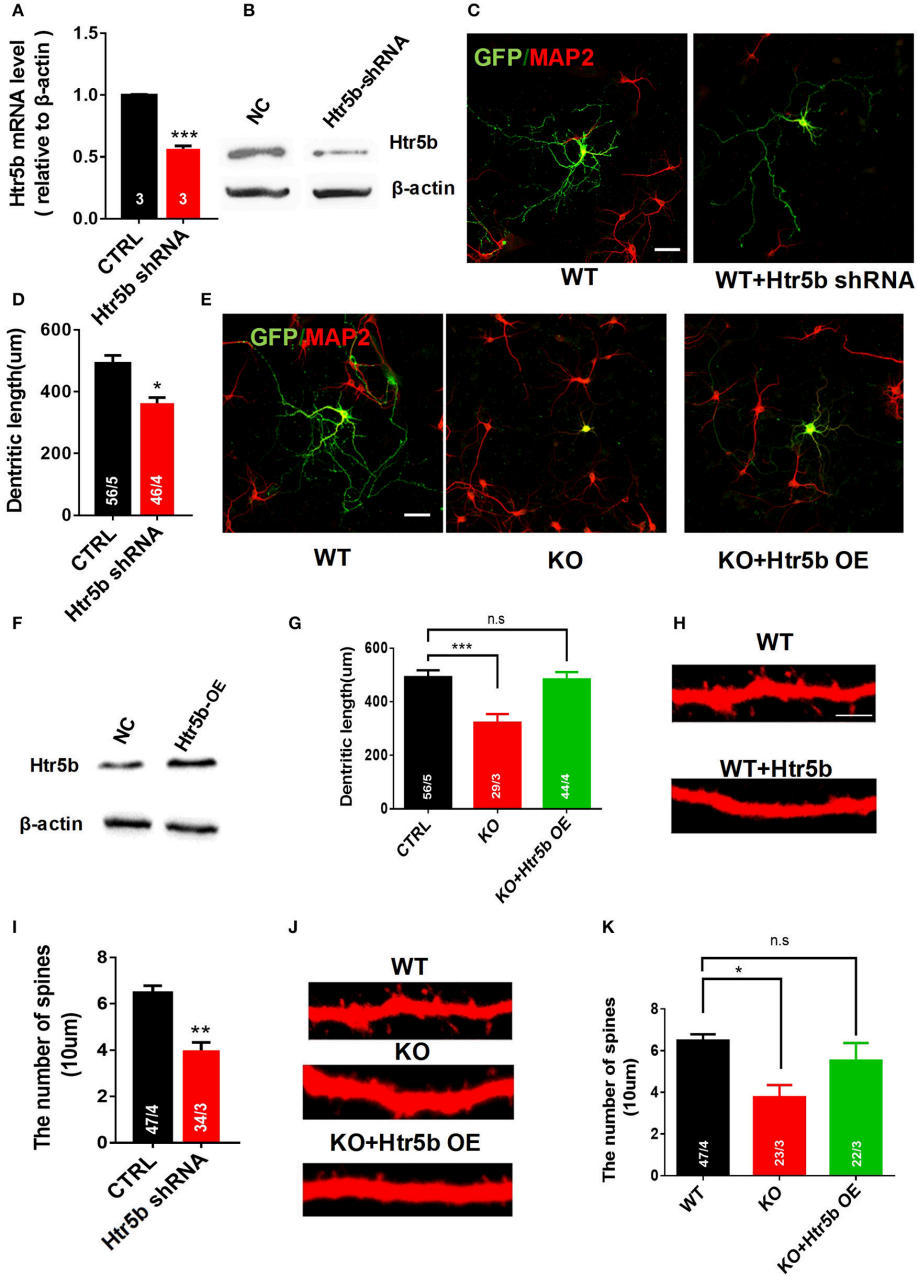
Restoring *Htr5b* in the hippocampal neurons of *Utx* cKO mice rescues morphological impairment. **(A)** *Htr5b* mRNA was decreased in newborn hippocampal neurons infected with Lenti-virus expressing *Htr5b* shRNA [Three independent experiments; two-tailed *t*-test: t_(4)_ = 18.03, *P* < 0.001]. **(B)** Western blotting for testing *Htr5b* protein levels in newborn hippocampal neurons infected with Lenti-virus expressing *Htr5b* shRNA. **(C)** Representative images of GFP (Green) and Map2 (Red) double positive neurons expressing either GFP or *Htr5b* shRNA in WT newborn hippocampal neurons (DIV7). Scale bars, 50 μm. **(D)** Quantification of dendritic length after *Htr5b* knockdown in WT newborn hippocampal neurons (DIV7) [WT, 56 neurons from 5 mice, cKO, 46 neurons from 4 mice; two-tailed *t*-test: t_(7)_ = 2.487, *P* = 0.0418]. **(E)** Representative images of GFP (Green) and Map2 (Red) double positive neurons expressing either GFP or *Htr5b* in WT or cKO newborn hippocampal neurons (DIV7). Scale bars, 50 μm. **(F)** Western blotting for testing *Htr5b* protein levels in newborn hippocampal neurons infected with Lenti-*Htr5b* overexpression virus. **(G)** *Htr5b* overexpression rescued the deficits of the dendritic length in cKO newborn hippocampal neurons (DIV7) [WT, 56 neurons from 5 mice; cKO, 29 neurons from 3 mice; KO+Htr5b, 44 neurons from 4 mice; One-way ANOVA: *F*_(2, 9)_ = 14.17, *P* = 0.0017]. **(H)** Representative images of spines from Map2 stained secondary dendrites of WT and cKO P0 hippocampal neurons (DIV19) expressing either GFP or *Htr5b* protein. Scale bars, 2 μm. **(I)** *Htr5b* knockdown resulted in the lower dendritic spine density in newborn hippocampal neurons (DIV7). **(J)** Representative images of spines from Map2 stained secondary dendrites of WT and cKO hippocampal neurons (DIV19) expressing either GFP or exogenous *Htr5b* protein. Scale bars, 2 μm. **(K)** *Htr5b* overexpression reversed the lower dendritic spine density in cKO neurons to similar levels of WT neurons (DIV19). Scale bars, 2 μm. ^*^P < 0.05, ^**^P < 0.01, ^***^P < 0.001. Error bars, s.e.m.

The serotonergic system is involved in many aspects of neural development, including neurite outgrowth, somatic morphology regulation, synaptogenesis, and control of dendritic spine shape and density (Wirth et al., 2016). To examine a functional relationship between *Utx* and *Htr5b* in mediating neural development, newborn hippocampal neurons from WT mice were transfected with lentivirus expressing both GFP and *Htr5b* shRNA sequences to suppress *Htr5b* at both mRNA and protein levels (Figures 8A,B). Knockdown of *Htr5b* resulted in a significant reduction in total dendritic length (Figures 8C,D) and spine density (Figures 8H,I). Given the fact that the expression level of *Htr5b* is decreased in *Utx* cKO mice that displayed neuronal morphological abnormalities (Figures 7E,F), we reasoned whether *Htr5b* gain-of-function could rescue neural development deficits in *Utx* cKO neurons. To test this assumption, exogenous *Htr5b* was expressed in *Utx* cKO newborn hippocampal neurons. Interestingly, *Htr5b* gain-of-function in cKO neurons was sufficient to restore the dendritic length (Figures 8E–G) and spine density (Figures 8J,K). These results suggest that *Htr5b* is a functional downstream target of *Utx* in modulating neuronal growth and morphology.

## Discussion

Recent human genetic findings indicate that epigenetic modifications, including histone methylation, are important regulators of neural development (Portela and Esteller, 2010). However, the underlying mechanisms remain largely unknown. Histone H3 lysine 27 demethylase *Utx* has been identified as a Kabuki syndrome-risk gene in patients with intellectual disability (Lederer et al., 2012; Miyake et al., 2013). Here, we demonstrate for the first time that forebrain deletion of *Utx* in mice leads to anxiety-like behaviors, spatial learning, and memory impairments. We further demonstrated that *Utx* deficiency impairs neuronal morphology and hippocampal synaptic transmission, providing novel evidence for the involvement of *Utx* in neural development and cognitive behaviors.

Regulation of neuronal development, such as dendrite and spine development, in the brain is critically important in a variety of physiological and pathological conditions (Hering and Sheng, 2001; de la Torre-Ubieta and Bonni, 2011). For instance, mental disorders and alterations in learning and memory are frequently accompanied by analogous modifications of dendrite and spine (Beique et al., 2006). In the present study, we provided evidence for the first time both *in vivo* and *in vitro* that UTX is involved in the regulation of neuronal morphogenesis, including spine density and dendrite complexity. In particular, specific deletion of *Utx* in embryonic nervous system or down-regulation of *Utx* in adult hippocampus caused abnormal behavioral phenotypes, including anxiety-like behaviors and impairments in learning and memory, which may underscore congenital anomalies in Kabuki syndrome patients. Our study might shed new light on the understanding of epigenetic regulation, especially histone modification in neural development and cognitive behaviors. Since *Utx* is also expressed in other cell types (Smith et al., 2014), it would be interesting to explore if loss of *Utx* demethylase activity in non-neuronal cells also contributes to impairments in mood and cognition, as observed in *Utx* cKO mice.

LTP is widely considered to represent a cellular mechanism for the formation of specific types of anxiety-related behaviors, learning, and memory (Lisman and Raghavachari, 2006). Impaired LTP has been found in several anxiety animal models (Bannerman et al., 2014). In agreement with our behavioral results, we found that deletion of *Utx* significantly impairs LTP at CA3-CA1 synapses in the hippocampus, further confirming that *Utx* is an important modulator for neuronal behaviors. Cognitive abnormalities are closely correlated with altered synaptic transmission and plasticity in the hippocampus (Bannerman et al., 2014). AMPA-mediated mEPSCs are widely used to determine pre- and/or postsynaptic contribution to synaptic transmission and plasticity (Bekkers and Stevens, 1990). *Utx* cKO mice showed a decrease in the amplitude, but not the frequency, of mEPSCs, suggesting a postsynaptic defect in hippocampus (Xiao et al., 2007). Consistent with this finding, we observed a significant reduction of PSD-95, a key player in postsynaptic transmission (Beique et al., 2006). Our results suggest that impaired LTP and reduced amplitude of mEPSCs after *Utx* deletion lead to synaptic dysfunction, which, in turn, causes cognitive deficits in *Utx* cKO mice.

We observed that a null mutation of *Utx* caused mid-gestational lethality, which is consistent with the previous reports by others (Shpargel et al., 2012; Welstead et al., 2012). Male *Utx*-null mice can escape embryonic lethality, suggesting that this is likely due to the expression of *Uty*, a paralog that lacks H3K27 demethylase activity (Shpargel et al., 2012; Wang et al., 2012). However, the behavioral abnormalities observed in male *Utx*-null mice appeared not to be associated with *Uty* since we did not find any changes in *Uty* expression after *Utx* deletion in the brain, suggesting that *Uty* may not functionally compensate for *Utx* during neural development. In consistent with our observations, a recent study also showed that UTY cannot compensate for certain demethylase-dependent activities of UTX in T cell acute lymphoblastic leukemia (T-ALL) (Van der Meulen et al., 2015). Further studies in molecular and behavioral levels will be required to fully understand why male *Utx*-null mice can escape embryonic lethality and what functional role of *Uty* plays in the brain.

The involvement of the serotonin system in neural development has been well-established (Sparta and Stuber, 2014). Most serotonin receptor family members are known for their roles in mediating morphogenic signaling in neurons (Wirth et al., 2016) and cognition (Meneses, 1999). For example, several studies showed that *Htr3* promotes neurite outgrowth in thalamic neurons as well as in PC12 cells (Homma et al., 2006). Serotonin 3A receptor is involved in learning, cognition, and emotion (Gatt et al., 2010). Our transcriptional profiling RT-PCR results revealed that expression of *Htr5b*, a calcium signaling related gene encoding serotonin 5B receptor, is decreased in the hippocampus of *Utx* cKO mice. While *Htr5b* is highly expressed in CNS (Meneses, 1999), the signaling pathways regulated by *Htr5b in vivo* are still not well-understood. This is largely due to the lack of proper receptor ligands (Wirth et al., 2016). More recently, there is evidence showing that *Htr5b* is a downstream target for transcription factor ATF-7, which mediates abnormal behaviors in mice (Maekawa et al., 2010). In that study, authors observed that ATF-7-deficient mice exhibit abnormal behaviors and increased *Htr5b* mRNA levels in the dorsal raphe nuclei (Maekawa et al., 2010). In the present study, we observed that knockout of *Utx* led to down-regulation of *Htr5b* and displaying anxiety-like behaviors and spatial learning and memory disability, suggesting that a balance in *Htr5b* expression levels would be critically important for maintaining the functional normality in the nervous system.

In addition to the observations made *in vivo*, we also found that down-regulation of *Htr5b* in cultured hippocampal neurons resulted in reduced dendritic length and spine density. Our results reveal a novel functional role of *Htr5b* in regulation of dendrite morphology development. Specifically, we observed that the deletion of *Utx* results in a decreased *Htr5b* associated with an increased H3K27me3 modification. ChIP assay showed high enrichment of H3K27me3 in the promoter region of *Htr5b*. These results suggest that regulation of *Utx* in *Htr5b* expression is likely mediated through demethylation of H3K27me3. Functionally, overexpression of *Htr5b* in cultured neurons could rescue the impairments in dendritic length and spine density induced by *Utx* deletion, suggesting that *Htr5b* is involved in UTX-mediated H3K27me3 de-methylation in regulation of neuronal morphology. Our findings provide a novel mechanism underlying the role of *Utx* in neural development, synaptic transmission, and cognition. Assessment of behavioral performance by modulating H3k27me3 levels in genetically animal models together with UTX inhibitors would provide more insights into the mechanisms underlying H3K27me3 modification in cognitive function, which remains to be studied.

In summary, we show here that H3K27 demethylase enzyme *Utx* loss-of-function contributes to impairments in neuronal development and synaptic plasticity, which are responsible for mood and cognitive deficits in *Utx* cKO mice. Given the fact that behavior changes in *Utx* loss-of-function mice replicate some symptoms in human Kabuki patients, *Utx* cKO mouse models like ours therefore provide a valuable means to study the underlying mechanisms of the etiology of Kabuki syndrome, and to develop novel clinical implications.

## Significance statement

Trimethylation of histone H3 lysine 27 (H3K27me3) establishes a repressive chromatin state in silencing gene expression. The demethylases UTX mediates the removal of H3K27me2/3 to establish a mechanistic switch to activate large sets of genes. Moreover, defects in UTX cause Kabuki syndrome characterized by congenital anomaly and mental retardation. We discovered that deletion of UTX in the brain results in increased anxiety-like behaviors, impaired spatial learning and memory, and neuronal morphology deficiency in mice. UTX regulates a subset of genes that are associated with dendritic morphology, synaptic transmission, and cognition. This study enhances our understanding the cognitive and developmental deficits in Kabuki syndrome.

## Author contributions

GT, ZT, and CL designed research; GT, YZ, and PL performed the majority of the experiments; TM and LY performed the electrophysiological recordings; SZ, SD, QT, YX, HY, and HD contributed to collection and assembly of data; GT, ZT, and CL wrote the paper; CL, FZ, and ZT supervised the research.

### Conflict of interest statement

The authors declare that the research was conducted in the absence of any commercial or financial relationships that could be construed as a potential conflict of interest.
